# Crosslink: An R Package for Network Visualization of Grouped Nodes

**DOI:** 10.3389/fgene.2021.706854

**Published:** 2021-07-16

**Authors:** Di Liu, Zhijie Bai, Bing Liu, Zongcheng Li

**Affiliations:** ^1^Peking-Tsinghua Center for Life Sciences, Peking University, Beijing, China; ^2^State Key Laboratory of Proteomics, Academy of Military Medical Sciences, Academy of Military Sciences, Beijing, China; ^3^State Key Laboratory of Experimental Hematology, Institute of Hematology, Fifth Medical Center of Chinese PLA General Hospital, Beijing, China; ^4^Key Laboratory for Regenerative Medicine of Ministry of Education, Institute of Hematology, School of Medicine, Jinan University, Guangzhou, China

**Keywords:** R package, network, visualization, grouped data, crosslink

## Abstract

The demand for network visualization of relationships between nodes attributed to different categories grows in various biomedical research scenarios, such as gene regulatory networks, drug-target networks, ligand-receptor interactions and association networks of multi-omics elements. Elegantly visualizing the relationships between nodes with complex metadata of nodes and edges appended may inspire new insights. Here, we developed the crosslink R package, tailored for network visualization of grouped nodes, to provide a series of flexible functions for generating network diagrams. We first designed a CrossLink class for storage of metadata about nodes and edges and manipulation of node coordinates. Then affine transformation and function mapping transformation are implemented to perform fundamental node coordinates transformation by groups, based on which various network layouts can be defined easily. For convenience, we predefined several commonly used layouts, including row, column, arc, polygon and hive, which also can be combined in one layout. Finally, we designed a user-friendly wrapper function to draw network connections, aesthetic mappings of metadata and decoration with related annotation graphs in one interface by taking advantage of the powerful ggplot2 system. Overall, the crosslink R package is easy-to-use for achieving complex visualization of a network diagram of grouped nodes surrounded by associated annotation graphs.

**Availability and Implementation:**

Cosslink is an open-source R package, freely available from github: https://github.com/zzwch/crosslink; A detailed user documentation can be found in https://zzwch.github.io/crosslink/.

## Introduction

With the rapid development of multi-omic technologies, intricate relationships between different categories of *biomedical* molecules were established, which brought huge opportunities and challenges to network visualization. Visualization of relationships between various biomolecules from different layers is helpful to explain and extract comprehensive biological information. For instance, Youqiong Ye etc. presented the network among the identified molecular alterations and the sensitivity of anticancer drugs to directly display a multi-omic molecular feature landscape of tumor hypoxia (Ye et al., 2019). And recently, there is a study characterizing the network among the expression of altered m6A regulators and cancer related pathways to illustrate the role of m6A in carcinogenesis (Li et al., 2019). Besides, researches in brain disease and plant development often provide an intuitive correlation network diagram to explain the influence of key regulators on other related layers (Shahan et al., 2018; Gilson et al., 2020). These cases show the common elements required for network visualization in many *biomedical* researches: (1) connections between multiple groups of biomolecules (i.e., grouped nodes), (2) mapping of additional biological information onto biomolecules and connections (i.e., nodes and edges), (3) arrangement of biomolecules in columns according to their categories, and (4) combination of annotation graphs around the network diagram.

A number of tools have been developed for visualization of various complex network, such as Cytoscape (Shannon et al., 2003), igraph (Csardi and Nepusz, 2006), ggraph (Pedersen, 2020) and Gelphi (Bastian et al., 2009). Recently, CellChat (Jin et al., 2021) was released to specifically analyze and visualize cell-cell communication network. Importantly, none of the tools above offer the function to combine the network diagram with the corresponding annotation graphs for grouped nodes. For the present, a tool specially designed for network visualization of grouped nodes that supports nodes decoration with annotation plots is still lacking.

Therefore, the user-friendly R package crosslink is developed here to arrange nodes by group, map metadata onto aesthetics of nodes and edges and align annotation graphs with the network. This package would hopefully meet various specific demands on network visualization of grouped biomolecules in *biomedical* research.

## Materials and Methods

**FIGURE 1:**
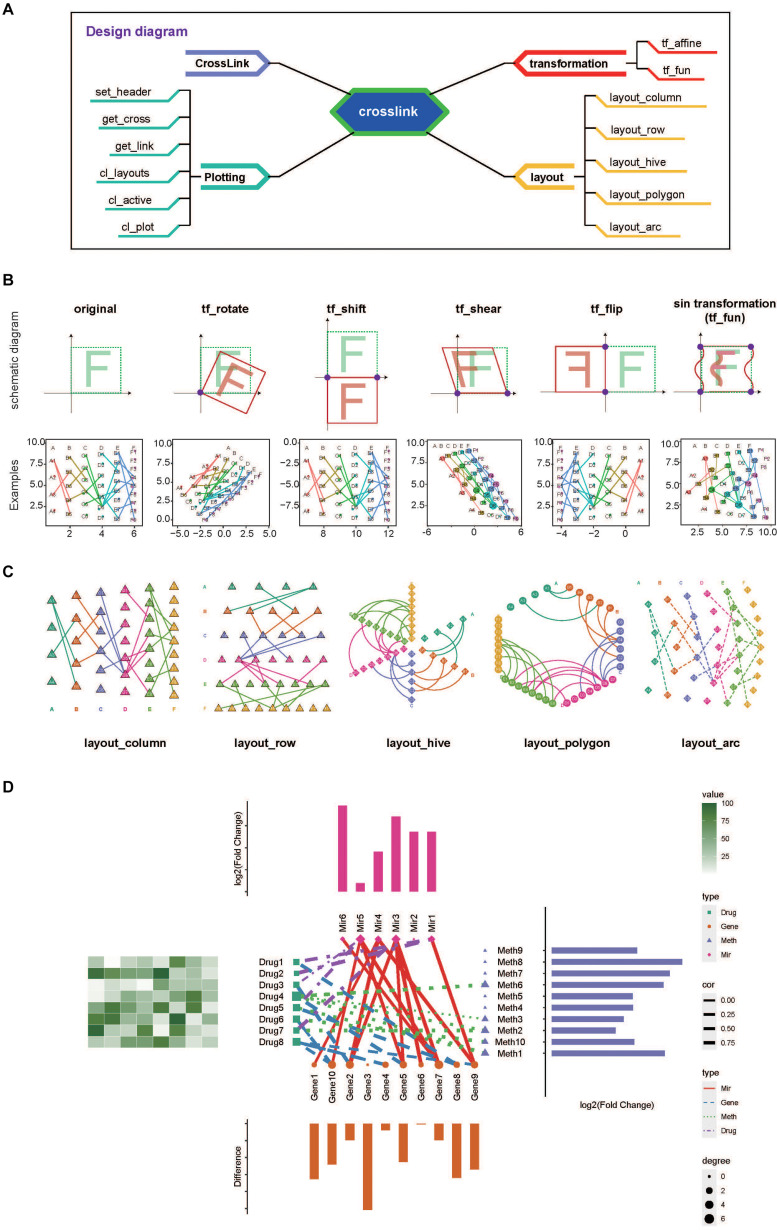
Overviw and usage examples of crosslink. **(A)**. A schematic diagram of crosslink showing four modules and associated functions. **(B)** Schematic diagram and examples showing transformation effects after using the coordinate transformation functions as indicated. **(C)** Examples of five predefined layout styles. **(D)** A typical application of combination network visualization by using crosslink.

The crosslink is developed in R language and mainly includes four modules, which is CrossLink class, coordinate transformation methods, layout modules and the plotting function, as shown in Figure 1A. The CrossLink class is the basic module, storing the metadata of nodes and edges, node coordinates and other parameters. The other three modules are operated on the data structure of CrossLink class. Here, we termed the group of nodes as “cross” and the edge between groups as “link”.

First, the function “crosslink” is used to generate a CrossLink object. With this function users can easily initialize a default network by inputting nodes and edges information. Several adjustments including spaces between nodes and gaps between crosses (groups) are also available for fine-tuning the default layout.

Second, coordinate transformation module, consisting of several affine transformation methods and the method to define the function for mapping transformation, is then applied for node coordinate transforming by crosses. The “tf_affine” function is designed for coordinate transforming of grouped nodes in the network. It requires a CrossLink object as the input and returns the object with transformed coordinates. This function provides several designed modes including rotating, shifting, shearing, flipping and scaling (Figure 1B), which would be useful when adjusting node coordinates in one or all groups to beautify presentation of complex relationships among multiple types of data, as shown in Figure 1D. The “tf_fun” interface allows users to customize transforming function according to specific needs. Here, as an example, we designed a “sin” transformation method using “tf_fun” interface to illustrate its usage (Figure 1B).

Third, *commonly used styles are predefined in the* layout module, *including row, column, arc, polygon and hive as shown in* Figure 1C. Users can specify a predefined network layout or combine multiple predefined layouts to design a diverse network.

Fourth, the plotting function “cl_plot” allows various aesthetic settings for nodes, edges, node labels and headers by taking advantage of “ggplot2” system (Ito and Murphy, 2013). In particular, this function provides the annotation interface to achieve the combination of the network diagram and corresponding annotation graphs, with node coordinates aligned (Figure 1D). Additionally, the plotting module also includes several data extraction functions, such as “get_cross” and “get_link”, which can be used to obtain the coordinate and metadata information. The “set_header” function is provided to place cross (group) headers.

In summary, crosslink provides a friendly interface for users to realize diverse network plotting of grouped nodes. This package can be applied to various biomedical studies for visualizing complex information and relationships between biomolecules in different categories (Goh et al., 2007; Neph et al., 2012; Chen and Wu, 2013; Shahan et al., 2018).

## Discussion

This work presented the first network visualization R package tailored for grouped nodes that implements a series of functions to store network data, manipulate node coordinates, and plot network diagram with supports for aesthetic mappings for nodes and edges and aligned graph annotation.

## Data Availability Statement

The original contributions presented in the study are included in the article/Supplementary Material, further inquiries can be directed to the corresponding author/s.

## Author Contributions

ZL and BL conceived and designed the study. ZL completed the R package “crosslink” and wrote the manuscript. DL performed the figure test and wrote the user guide and the manuscript. ZB proofread and corrected the manuscript. All authors contributed to the article and approved the submitted version.

## Conflict of Interest

The authors declare that the research was conducted in the absence of any commercial or financial relationships that could be construed as a potential conflict of interest.

## References

[B1] Bastian M. Sebastien H. Mathieu J. (2009). “Gephi: an open source software for exploring and manipulating networks,” in *Proceeding of the International AAAI Conference on Web and Social Media.*

[B2] Chen B. S. Wu C. C. (2013). Systems biology as an integrated platform for bioinformatics, systems synthetic biology, and systems metabolic engineering. *Cells* 2 635–688. 10.3390/cells2040635 24709875 PMC3972654

[B3] Csardi G. Nepusz T. (2006). The igraph software package for complex network research. *InterJ. Comp. Syst.* 1695. Available online at: https://igraph.org

[B4] Gilson M. Zamora-López G. Pallarés V. Adhikari M. H. Senden M. Campo A. T. (2020). Model-based whole-brain effective connectivity to study distributed cognition in health and disease. *Netw Neurosci.* 4 338–373. 10.1162/netn_a_00117 32537531 PMC7286310

[B5] Goh K. I. Cusick M. E. Valle D. Childs B. Vidal M. Barabási A. L. (2007). The human disease network. *Proc. Natl. Acad. Sci. U.S.A.* 104 8685–8690. 10.1073/pnas.0701361104 17502601 PMC1885563

[B6] Ito K. Murphy D. (2013). Application of ggplot2 to pharmacometric graphics. *CPT Pharmacometrics Syst. Pharmacol.* 2:e79. 10.1038/psp.2013.56 24132163 PMC3817376

[B7] Jin S. Guerrero-Juarez C. F. Zhang L. Chang I. Ramos R. Kuan C. H. (2021). Inference and analysis of cell-cell communication using CellChat. *Nat. Commun.* 12:1088. 10.1038/s41467-021-21246-9 33597522 PMC7889871

[B8] Li Y. Xiao J. Bai J. Tian Y. Qu Y. Chen X. (2019). Molecular characterization and clinical relevance of m(6)a regulators across 33 cancer types. *Mol. Cancer* 18:137. 10.1186/s12943-019-1066-3 31521193 PMC6744659

[B9] Neph S. Stergachis A. B. Reynolds A. Sandstrom R. Borenstein E. Stamatoyannopoulos J. A. (2012). Circuitry and dynamics of human transcription factor regulatory networks. *Cell* 150 1274–1286. 10.1016/j.cell.2012.04.040 22959076 PMC3679407

[B10] Pedersen T. L. (2020). *ggraph: An Implementation of Grammar of Graphics for Graphs and Networks.* R package version 2.0.4. Available online at: https://CRAN.R-project.org/package=ggraph

[B11] Shahan R. Zawora C. Wight H. Sittmann J. Wang W. Mount S. M. (2018). Consensus coexpression network analysis identifies key regulators of flower and fruit development in wild strawberry. *Plant Physiol.* 178 202–216. 10.1104/pp.18.00086 29991484 PMC6130042

[B12] Shannon P. Markiel A. Ozier O. Baliga N. S. Wang J. T. Ramage D. (2003). Cytoscape: a software environment for integrated models of biomolecular interaction networks. *Genome Res.* 13 2498–2504. 10.1101/gr.1239303 14597658 PMC403769

[B13] Ye Y. Hu Q. Chen H. Liang K. Yuan Y. Xiang Y. (2019). Characterization of hypoxia-associated molecular features to aid hypoxia-targeted therapy. *Nat. Metab* 1 431–444. 10.1038/s42255-019-0045-8 31984309 PMC6980239

