# Supplementary material for: Crosslink: An R Package for Network Visualization of Grouped Nodes
Source: Front Genet. 2021 Jul 16;12:706854. doi: 10.3389/fgene.2021.706854 (PMC8322777; doi:10.3389/fgene.2021.706854)
Supplement: Supplementary file 1 [file Presentation_1.PDF]

crosslink An R package for network visualization of grouped nodes

The goal of crosslink is to visualize the network of grouped nodes

1. Installation

You can install the released version of crosslink from github with:

```
remotes::install_github("zzwch/crosslink", build_vignettes = TRUE)
```

Or download the compressed file Link, then run

```
remotes::install_local(path = "./crosslink-master.zip", build_vignettes = TRUE)
```

2. Quick start

Examples of typical crosslink usage.

```
library(crosslink)

# generate a CrossLink object
cl <- crosslink(demo$nodes, demo$edges, demo$cross.by, odd.rm = F,spaces = "flank")

# set headers if needed
cl %<>% set_header(header = c("A","B","C","D","E","F"))

# plot the network

# By default, node color is coded and node size is proportional to its degree (calculated internally).
# And edge color is coded by the cross group of the edge's source node.
cl %>% cl_plot()
```

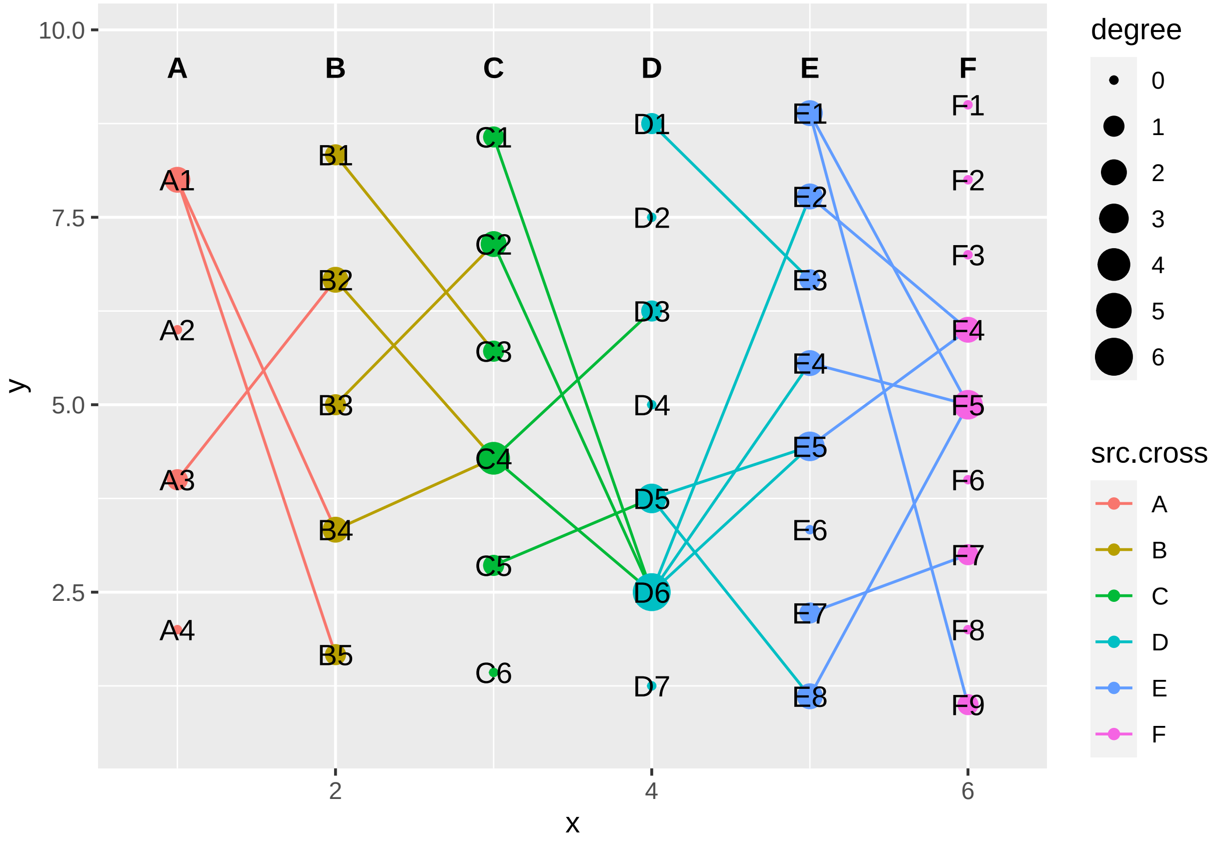

Users can also custom the aesthetics for node, edge, label and header by using cl_plot, which wrapped multiple ggplot2::geom_* functions in one interface. Please see below (Plotting modules) for more details.

3. Step by step

This is a basic example of the basic function of crosslink packages,including 1). Input data 2). Generate CrossLink class 3). Coordinate transformation 4). Layout modules 5). Plotting modules

1). Input data

crosslink needs two files as input.
a. nodes (must have two columns: node name and node type)
b. edges data (must have two columns: source node and target node).

Here, crosslink uses the function 'gen_demo' to generate demo data.

```
n <- 6
demo <- gen_demo(n_cross = n,n_node = 4:(n+3), n_link = 3:(n+1), seed = 66)
nodes <- demo$nodes
edges <- demo$edges
cross.by <- demo$cross.by
```

2). Generate crossLink class

crosslink can generate an object of crosslink class for plot.

```r
# users can define 'odd.rm' to choose if remove the nodes have zero relationship with any other nodes w
# user can set intervals between nodes and gaps through spaces and gaps.
cl <- crosslink(nodes, edges, cross.by, odd.rm = F,spaces = "flank")

# Header can be customized through 'set_header'
cl %<>% set_header(header = c("A","B","C","D","E","F"))
```

3). Coordinate transformation

The `default` layout is initialized by `crosslink` function.

```r
cl %>% cl_active() # get currrently active layout information of a CrossLink object
```

```
## [1] "default"
```

And, currently active layout will be based on for transforming coordinates. You can set another layout as active layout. All available layouts can be listed. See `layout modules` to set more layouts.

```r
cl %>% cl_layouts()  # get all available layouts in a CrossLink object
```

```
## [1] "default"
```

```r
cl_active(cl) <- "default" # set your favorate layout
```

Coordinate transformation consists of affine transformation and functional transformation. The `tf_affine` function contains `tf_rotate`, `tf_shift`, `tf_shear`, `tf_flip` and `tf_scale` function. The `tf_fun` interface allows user to custom transforming function.

Note : The active layout before transformation will be based on to perform transforming, and the transformed coordinates will be stored in 'transforming' layout (Default, set `layout` to change it, and a novel layout is permitted.).

```r
# tf_rotate, rotating in a specific angle with (x,y) as the center.
cl %>% tf_rotate(x=0,y=1,angle = 45) %>% cl_plot()
```

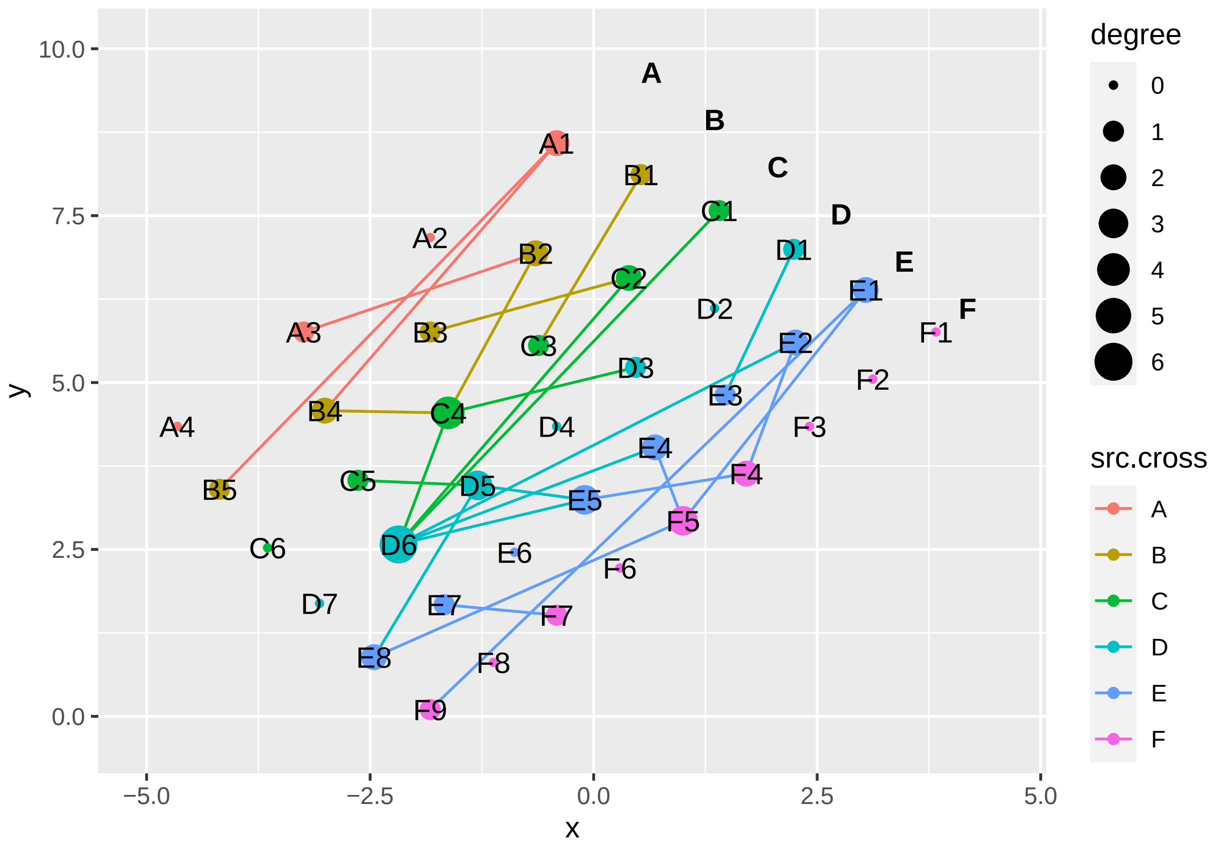


```r
# tf_shift, shifting a relative distance according to x-axis or y-axis
cl %>% tf_shift(x=1,y=-1) %>% cl_plot()
```

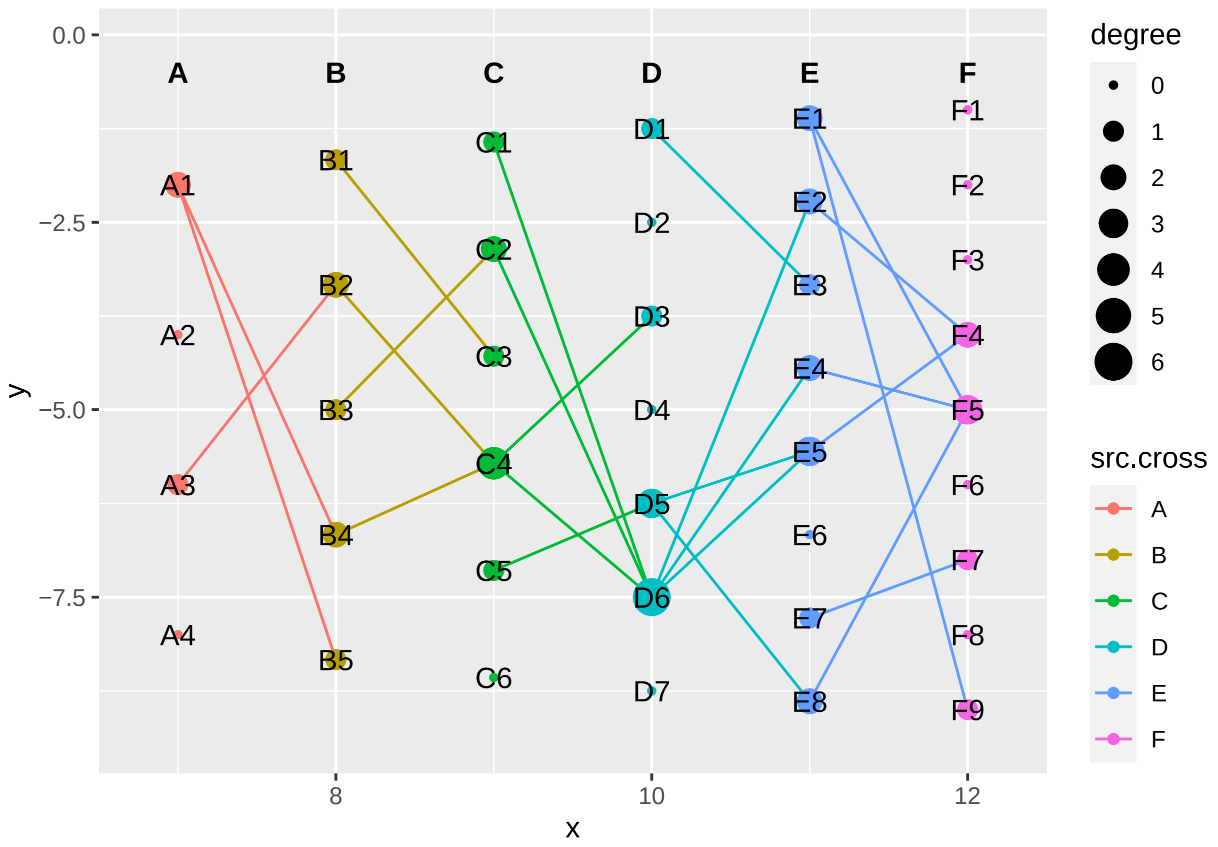


```r
# tf_shear
cl %>% tf_shear(axis = "x",angle = 60) %>% cl_plot()
```

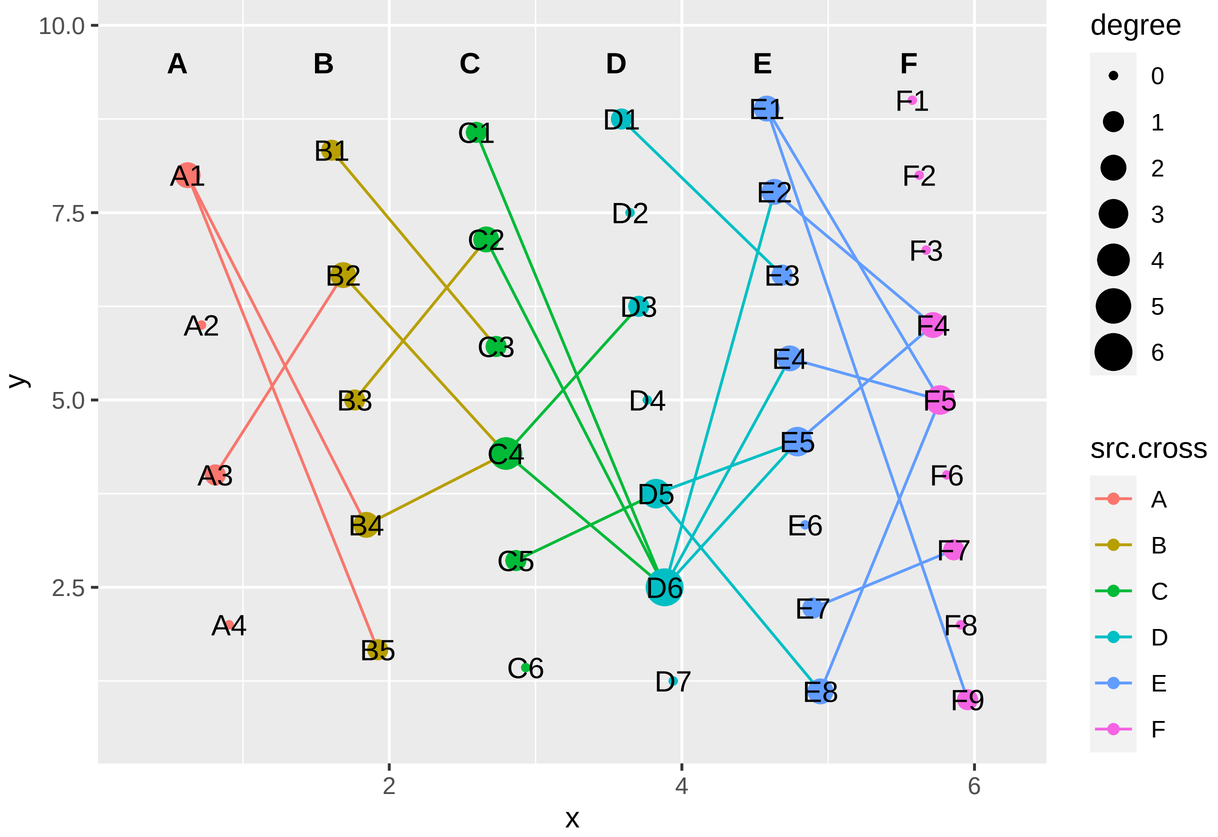


```
# tf_flip, flip the figure according to x-axis or y-axis
cl %>% tf_flip(axis = "y") %>% cl_plot()
```

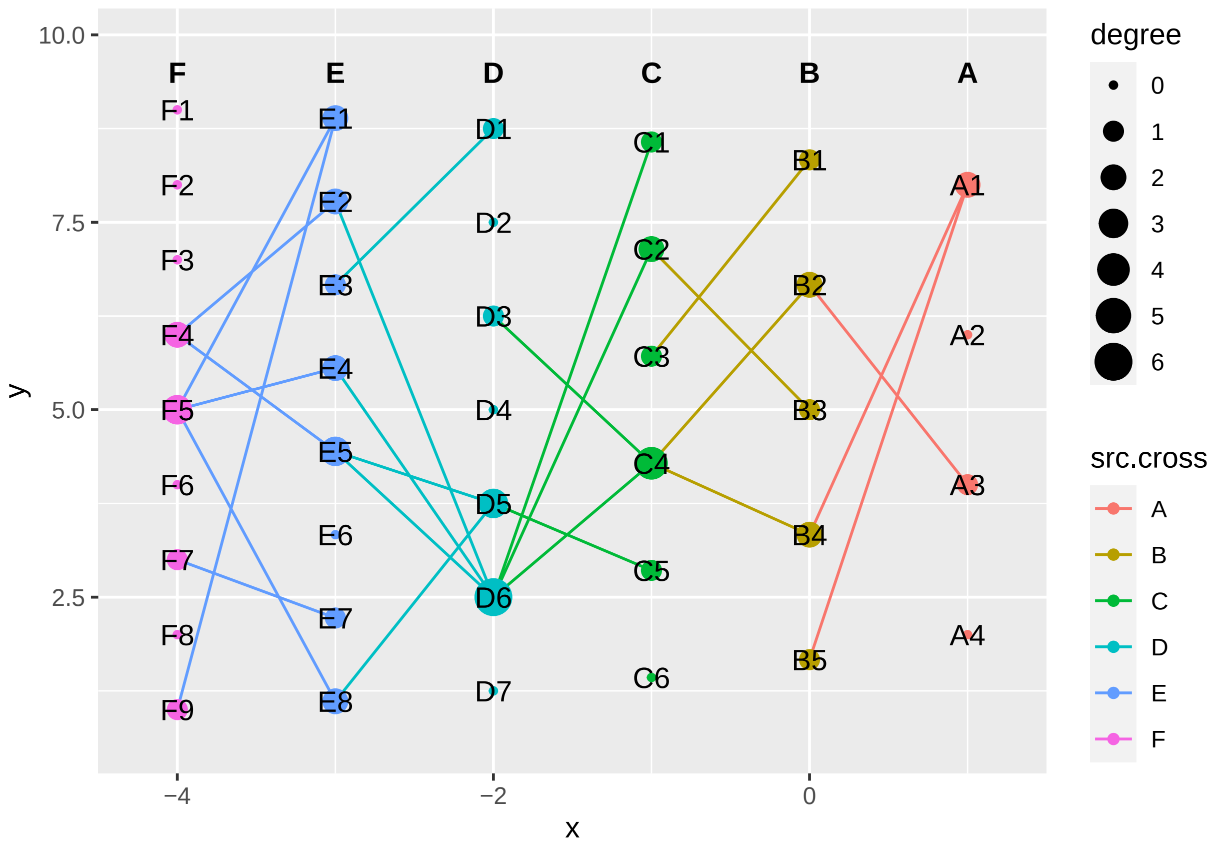


```r
# tf_scale
cl %>% tf_scale(x=0, y=1, scale.x = 5, scale.y = 5) %>% cl_plot()
```

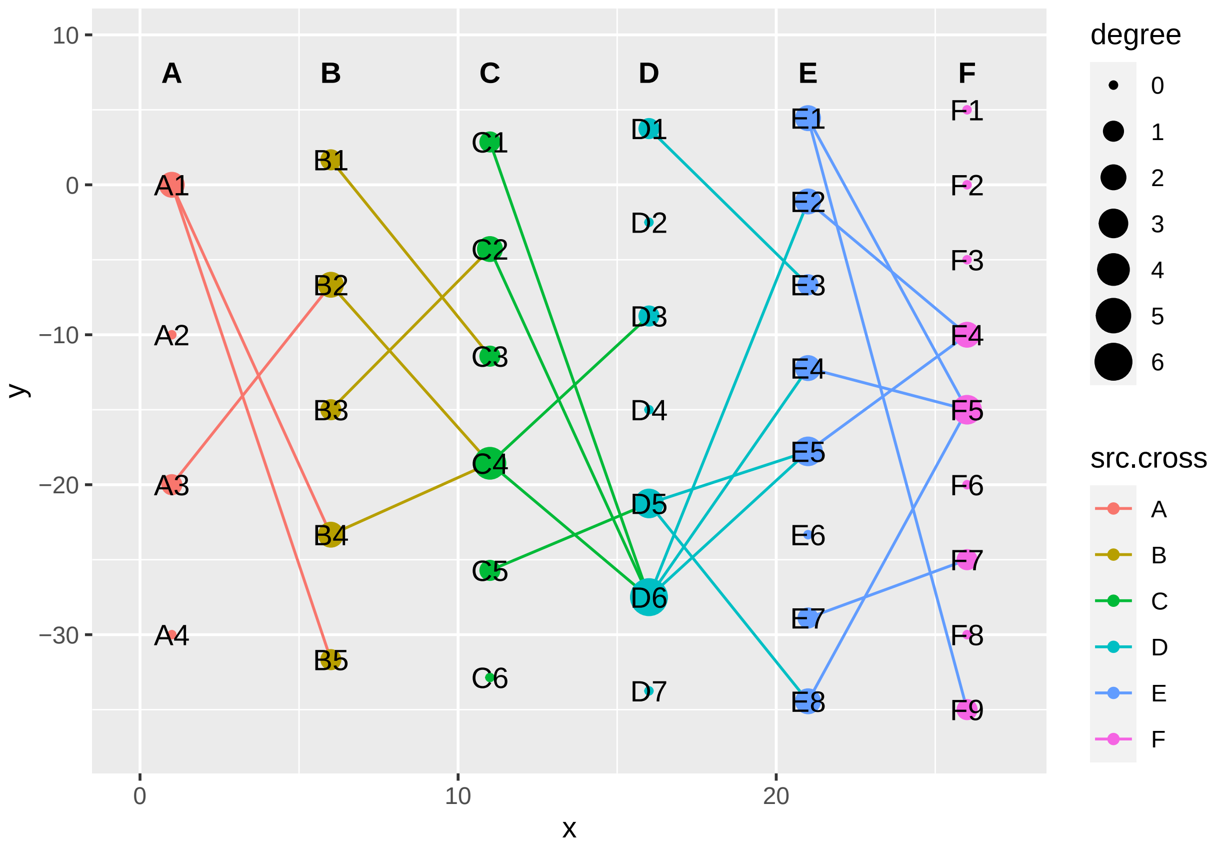


```
# tf_fun, coordinate transformation according to custom-defined function
cl %>% tf_fun(fun = sin,along = "y",xrange.from=c(0,0.5*pi)) %>% cl_plot()
```

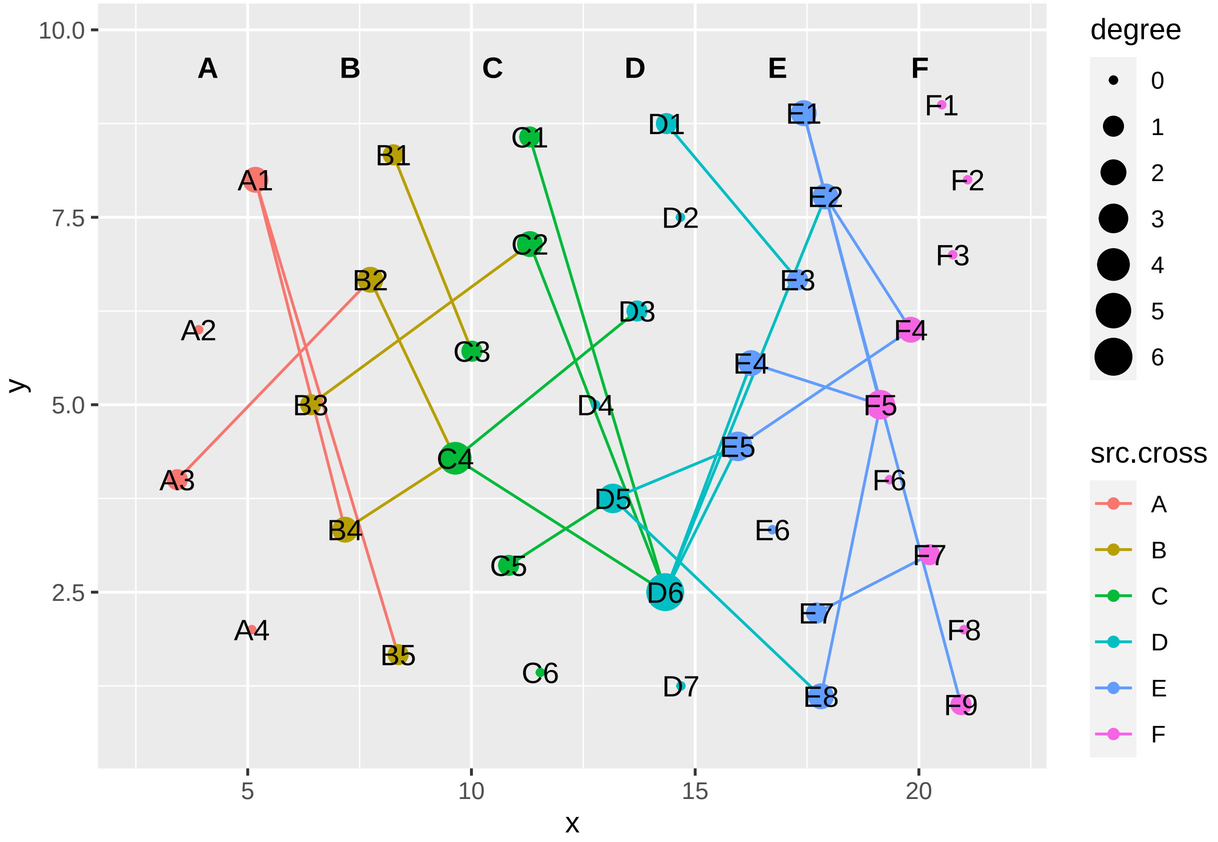


```
# combined transformation functions
cl %>% tf_flip(axis = "y") %>% tf_fun(fun = sin,along = "y",xrange.from=c(0,0.5*pi)) %>% cl_plot()
```

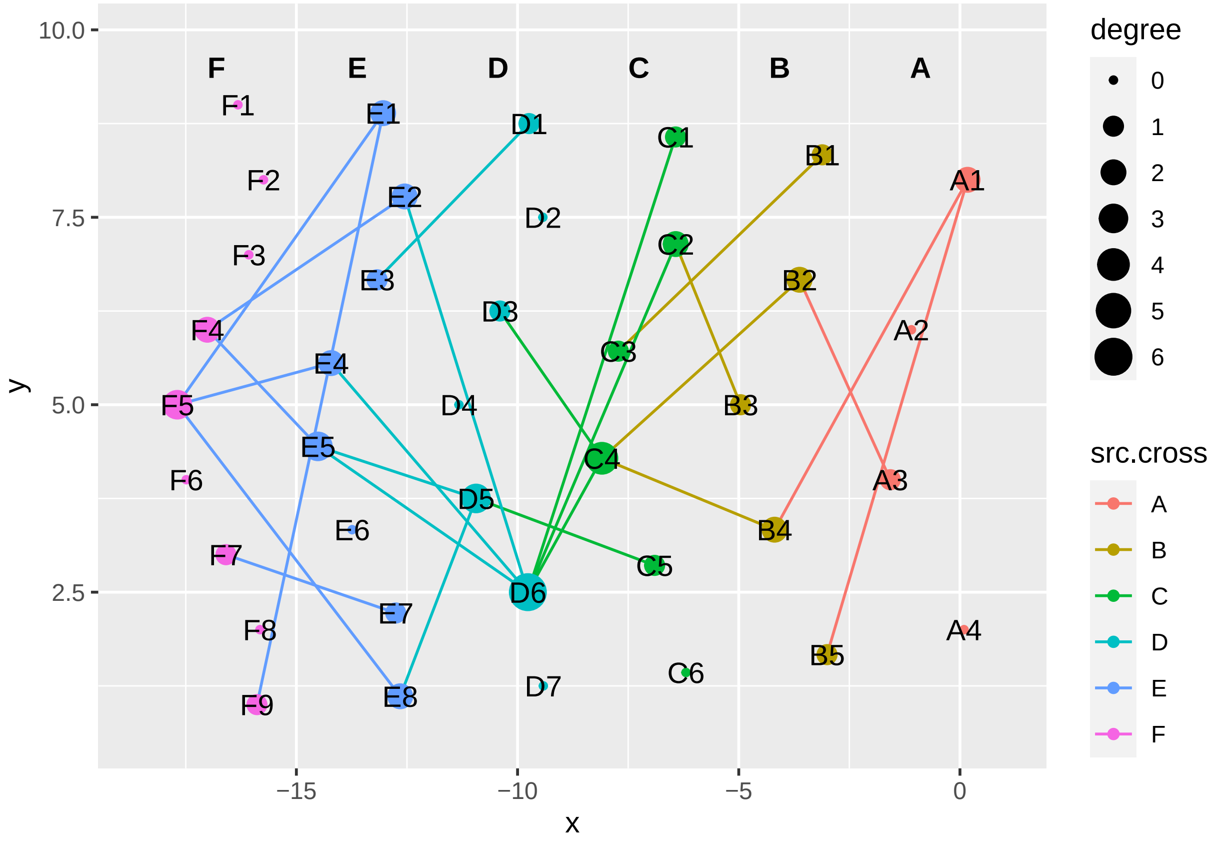

4). Layout modules

Different layout styles can be stored in a CrossLink object.

Because 'default' layout is routinely used as base layout for the layout module, it is strongly recommended not to override the 'default' layout (**Important**), unless you have known the transformation and layout modules well!

Several commonly used layout styles are predefined, including row, column, arc, polygon and hive. And crosses can be placed in one or multiple layouts.

Note : The 'set_header' function can be called to conveniently place headers after layouting.

```
# The 'default' layout is actually column.
cl %>% cl_plot()
```

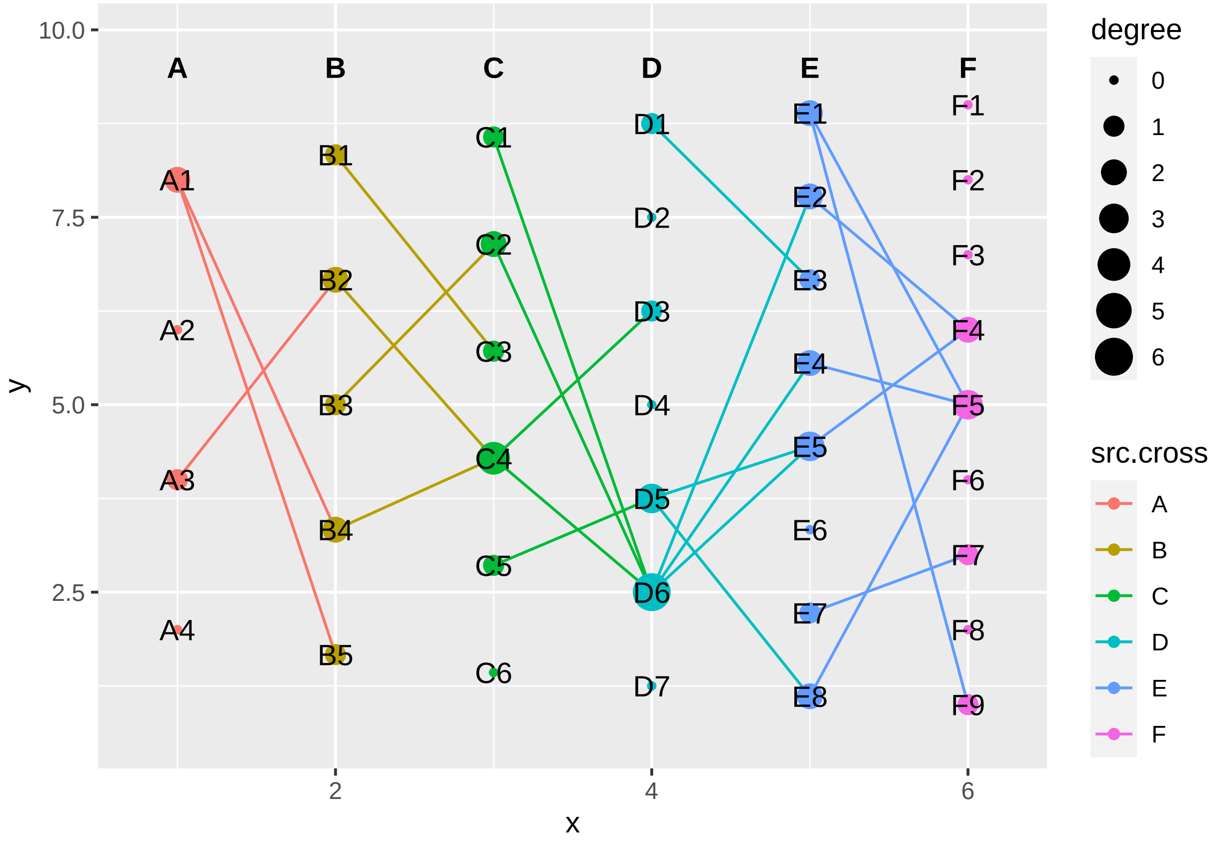


```
# layout by column
cl %>% layout_column(layout_save = "column") %>% cl_plot()
```

```
## Copy layout default into column, and Set active layout to column
## Copy layout default into column, and Set active layout to column
```

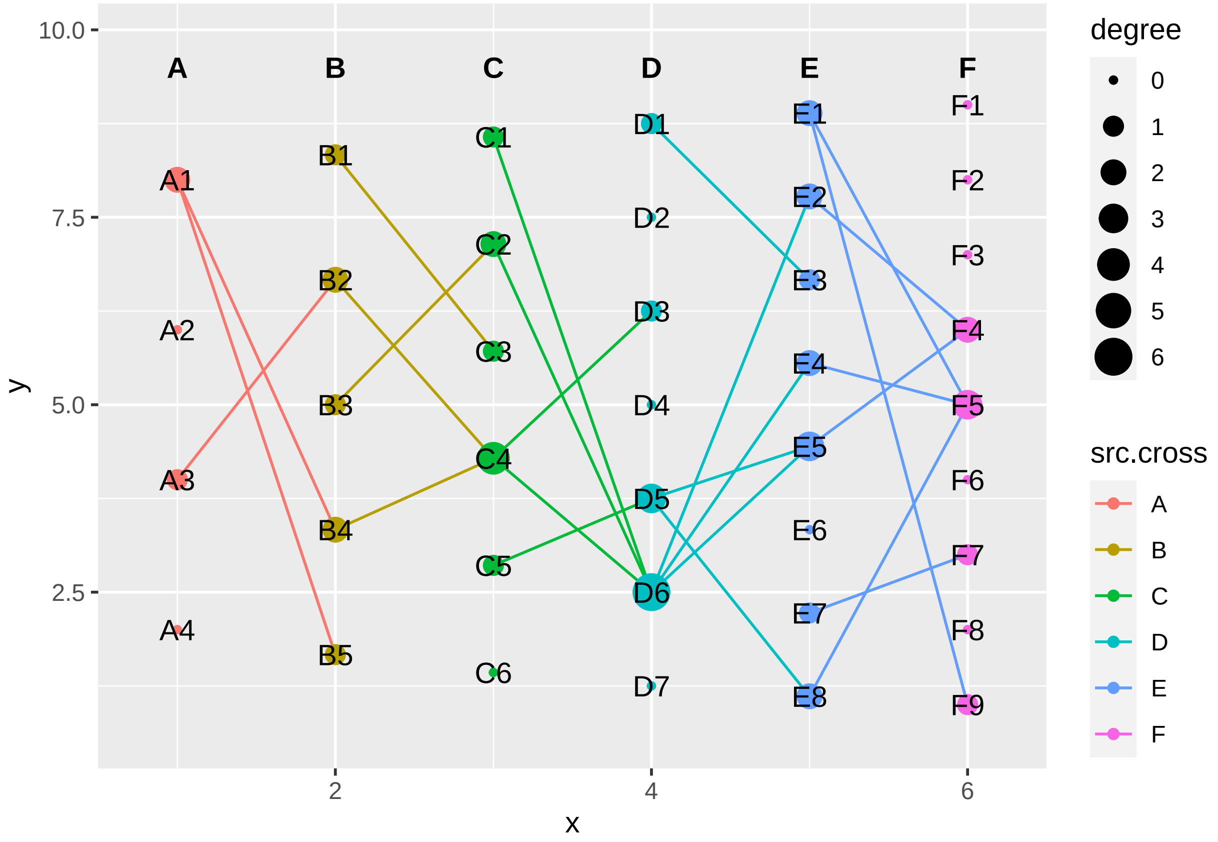


```
# layout by row
cl %>% layout_row(layout_save = "row") %>% cl_plot()
```

```
## Copy layout default into row, and Set active layout to row

## Copy layout temp into row, and Set active layout to row
```

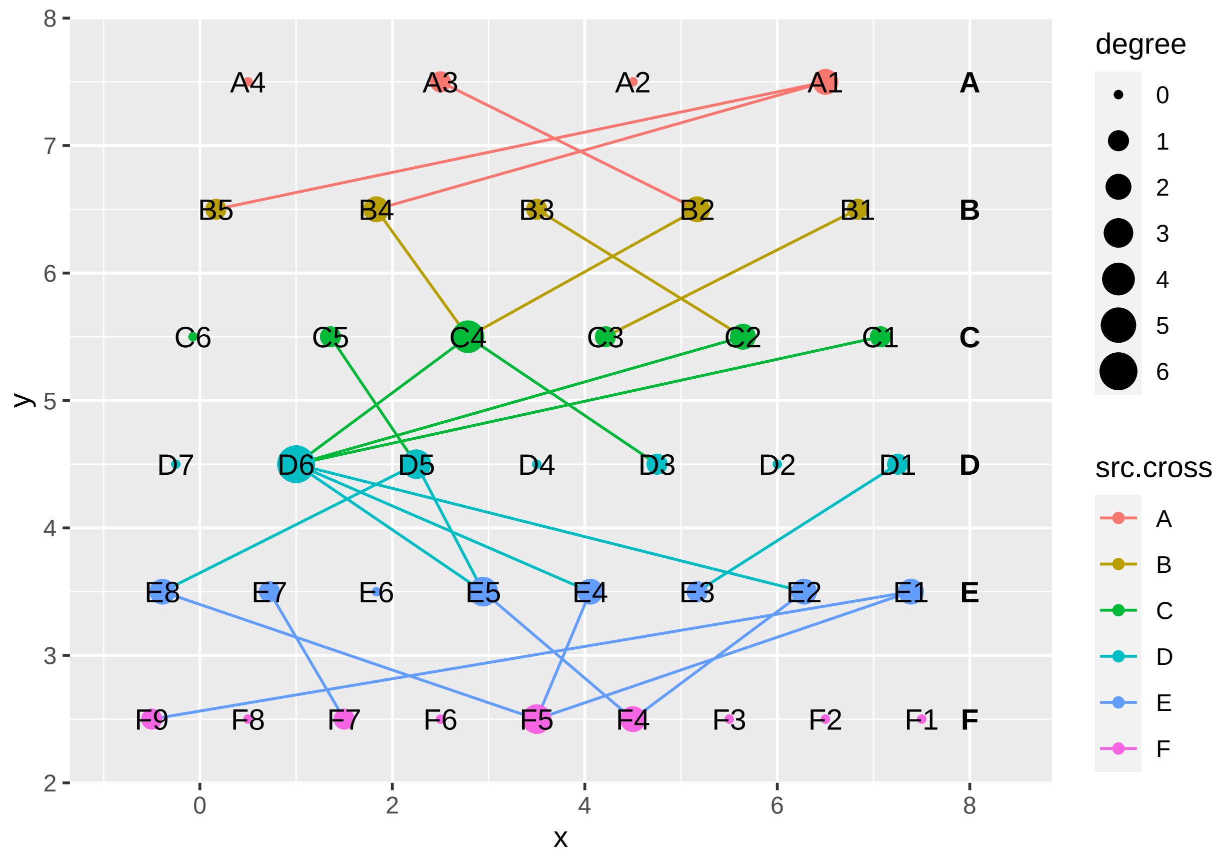


```
# layout by arc, set header after transformation
cl %>% layout_arc(angles = 60,crosses = c("E","F"), layout_save = "arc") %>% set_header(hjust = 0.5, vju
```

```
## Copy layout default into temp, and Set active layout to temp

## Copy layout default into arc, and Set active layout to arc

## Copy layout temp into arc, and Set active layout to arc
```

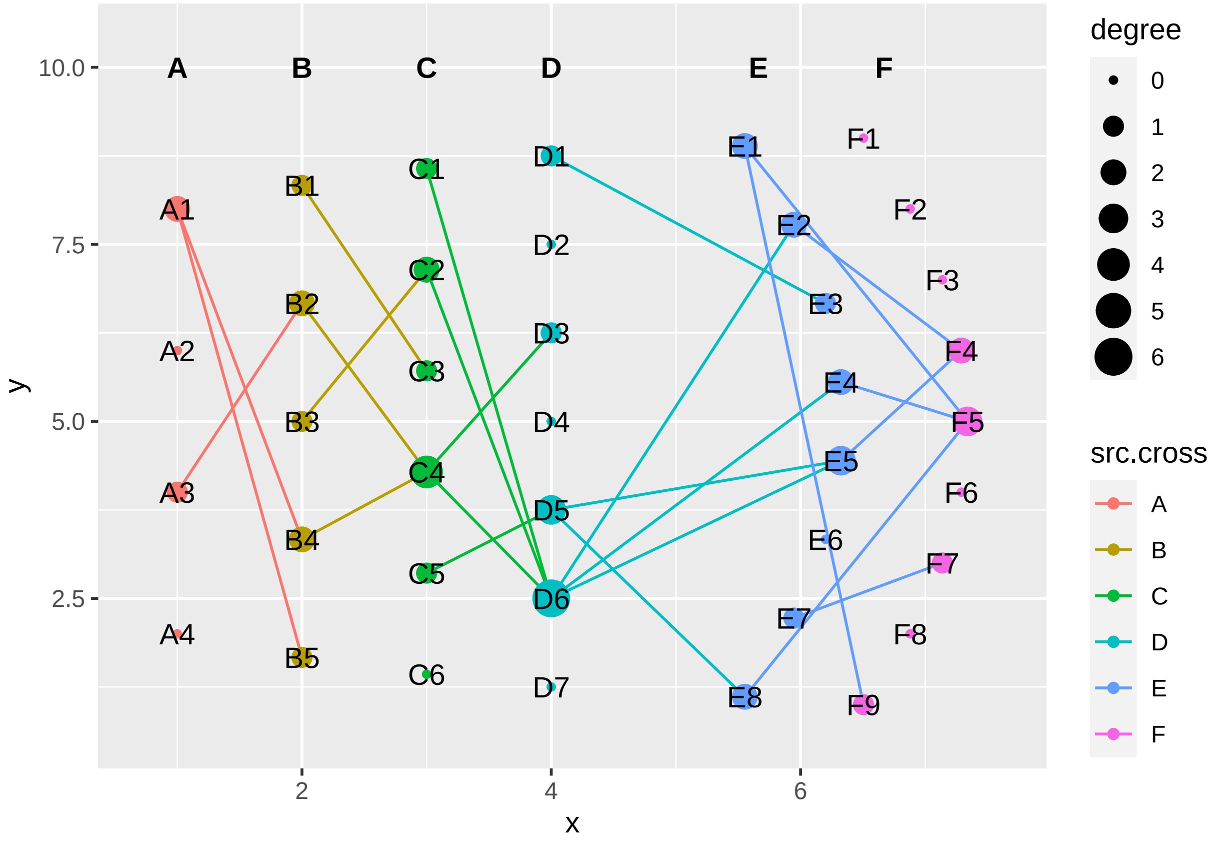


```r
# layout by polygon (list of angles must have the same length with crosses)
cl %>% layout_polygon(layout_save = "polygon") %>% cl_plot()
```

```
## Copy layout default into polygon, and Set active layout to polygon

## Copy layout temp into polygon, and Set active layout to polygon
```

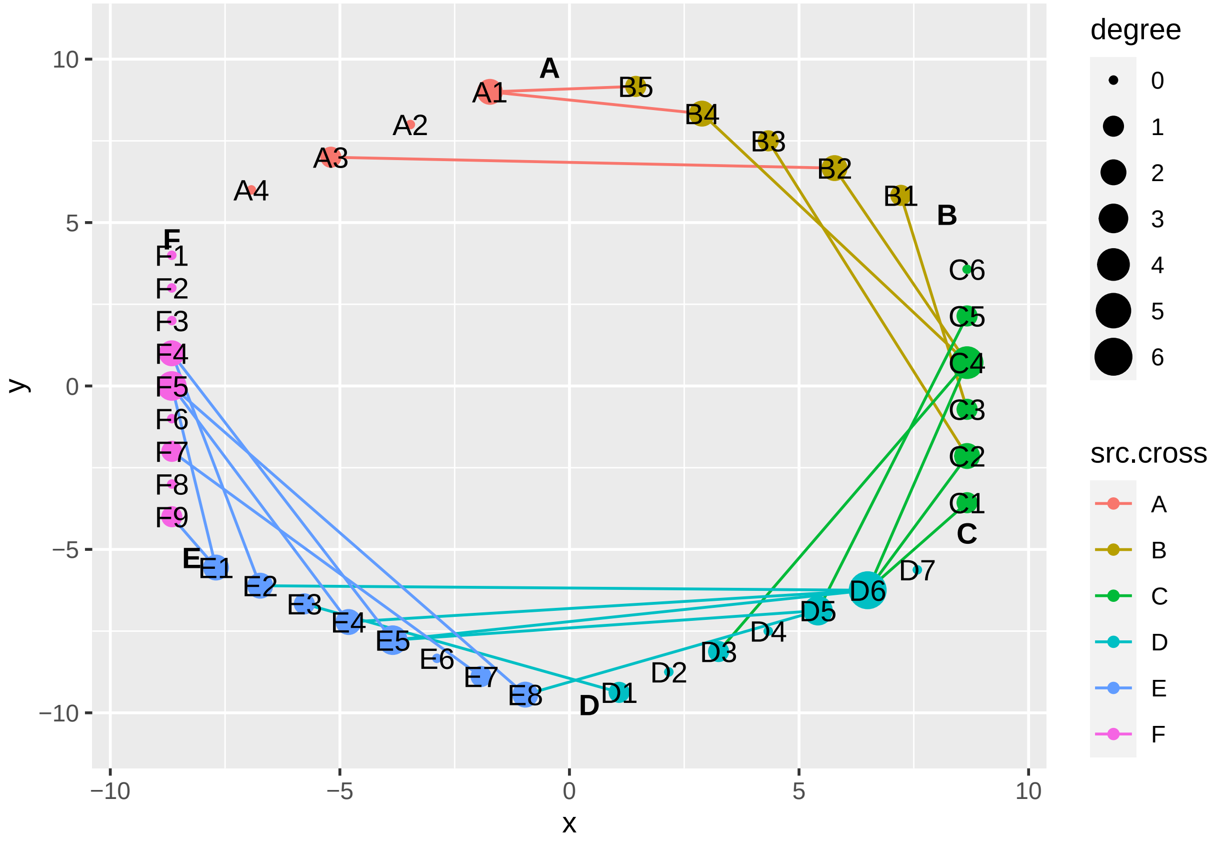


```r
# layout by hive
cl %>% layout_hive(layout_save = "hive") %>% cl_plot()
```

```
## Copy layout default into hive, and Set active layout to hive

## Copy layout temp into hive, and Set active layout to hive
```

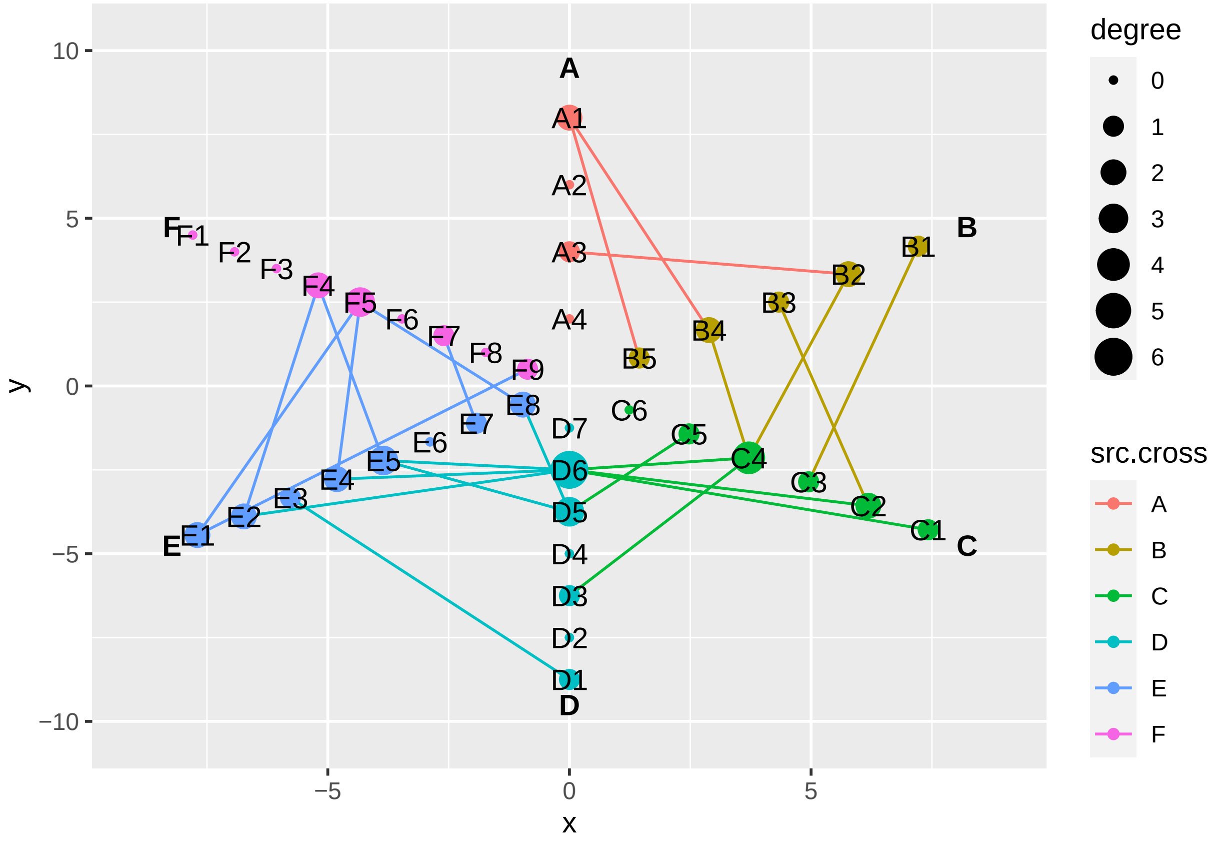

5). Plotting modules

We introduce this wrapper function `cl_plot` in three steps.

a. quick plotting (`cl_plot`)

b. aesthetic settings (The color, size, type and text of the nodes and lines in the network)

c. combination of the network diagram with the corresponding node annotation graph in aligning coordinates

a. quick plotting

```
cl %>% cl_plot()
```

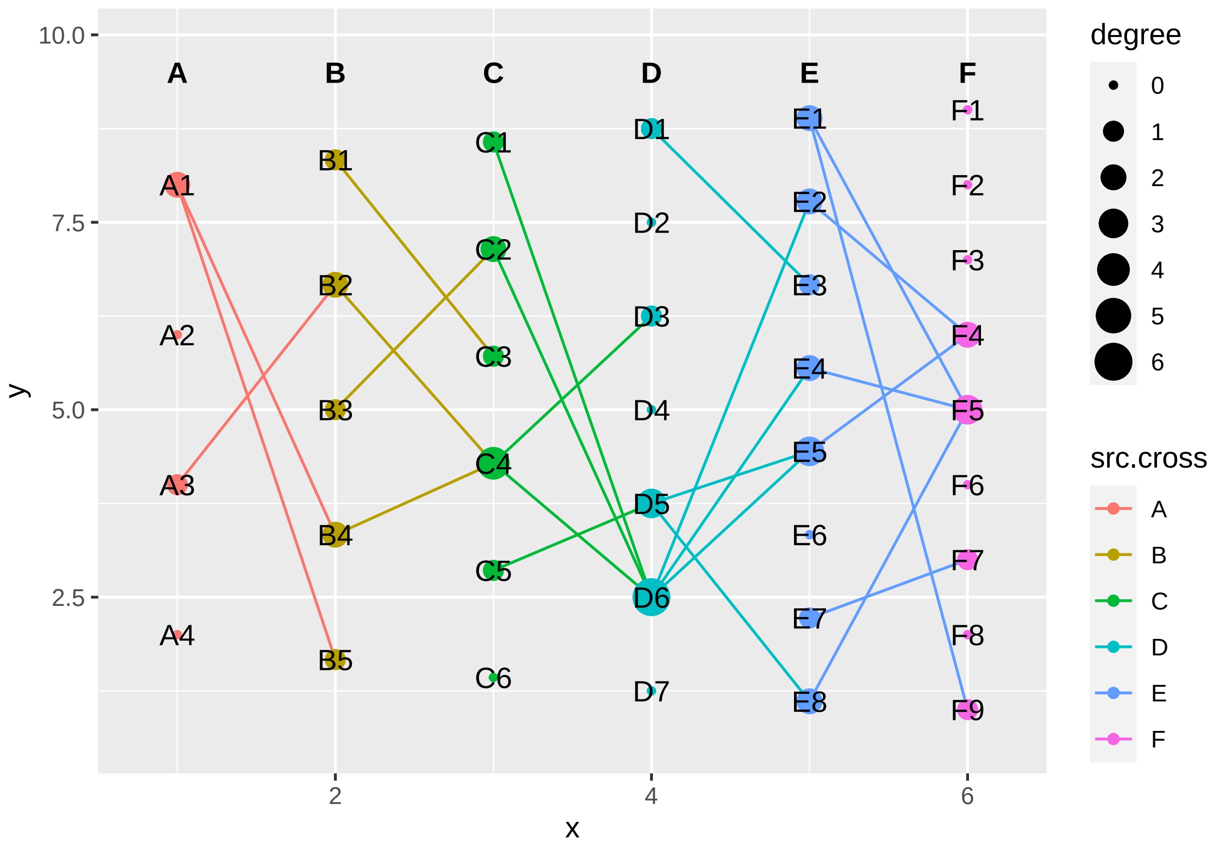

b. aesthetic settings

```
# aesthetic settings based on ggplot2 system, some specific examples are shown below.

# show available variables for aesthetic setting
cl %>% show_aes()
```

```
## Available meta.data names are showing below.
## Cross: node, node.type, x, y, cross, key, type, degree
## Link: src, tar, src.cross, tar.cross, source, target, src.degree, tar.degree, x, y, xend, yend
## Header: node, node.type, x, y, cross, header
```

```
# set colors, shapes and size of nodes
# cross:a named list of arguments for crosses. usage same as ggplot2::geom_point(). Set NULL to use def
cl %>% cl_plot(cross = list(mapping =  aes(color = type, shape= type),
                            scale   = list(color = scale_color_manual(values = RColorBrewer::brewer.pal
                                                                      guide = guide_legend(ncol = 2)),
                                           size  = scale_size_continuous(range = c(1,5),
                                                                      guide = guide_legend(ncol = 2)),
                                           shape  = scale_shape_manual(values = c(13:18),
                                                                      guide = guide_legend(ncol = 2))
                                          )
                           ))
```

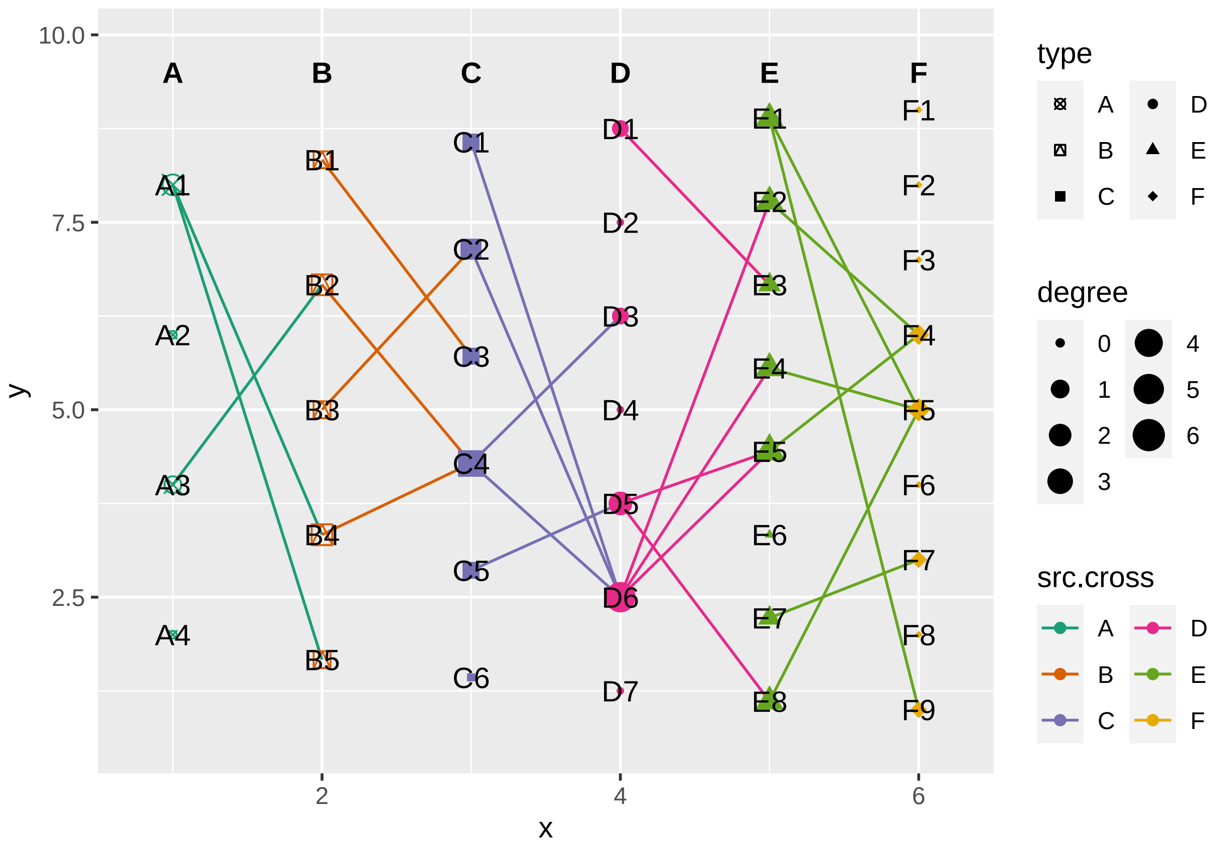


```
# set colors, linetypes and size of edges
# link: a named list of arguments for links. usage same as ggplot2::geom_segment(). Set NULL to use defa
cl %>% cl_plot(link = list(mapping =  aes(color = src.cross, linetype =src.cross, size = src.degree),
                           scale   = list(color = scale_color_manual(values = RColorBrewer::brewer.pal(5
                                                                     guide = guide_legend(ncol = 2)),
                                          size  = scale_size_continuous(range = c(1,3),
                                                                        guide = guide_legend(ncol = 2)),
                                          linetype = scale_linetype_manual(values = c(1:6),
                                                                     guide = guide_legend(ncol = 2))
                                          )
                          ),
               cross = list(show.legend = F) # disable cross's legends
               )
```

```
# set header styles
# header:   a named list of arguments for headers. usage same as ggplot2::geom_text(). Set NULL to use
cl %>% cl_plot(header = list(mapping = aes(color= cross),
                             scale = list(color = scale_color_manual(values = RColorBrewer::brewer.pal
                              size = 5.5
                            ))
```

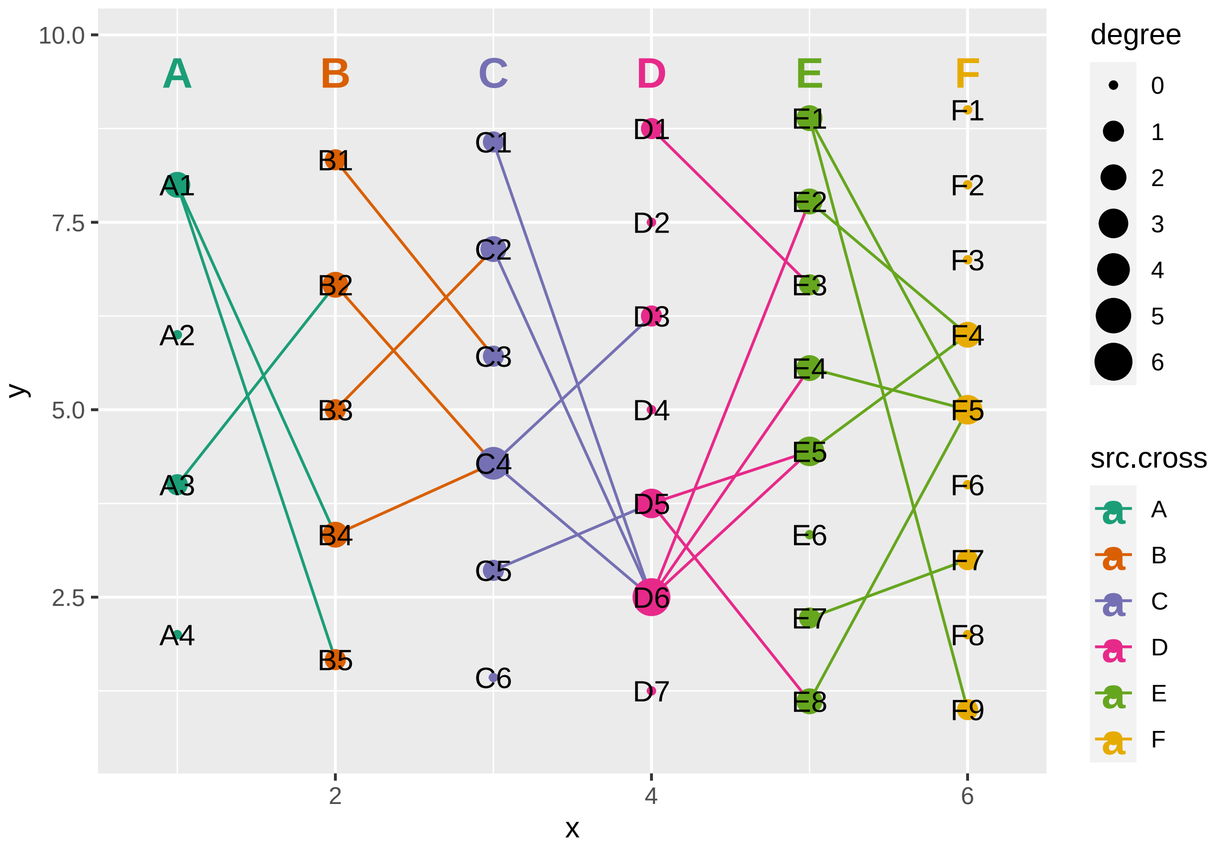


```
# set aesthetics (e.g., color, size, position) of labels
# label: a named list of arguments for labels of nodes. usage same as ggplot2::geom_text(). Set NULL to
cl %>% cl_plot(label = list(mapping =  aes(color = type),
                            scale   = list(color = scale_color_manual(values = RColorBrewer::brewer.pal
                                                                      guide = guide_legend(ncol = 2))
                                          ),
                            nudge_y = -0.3, size = 4
                           ))
```

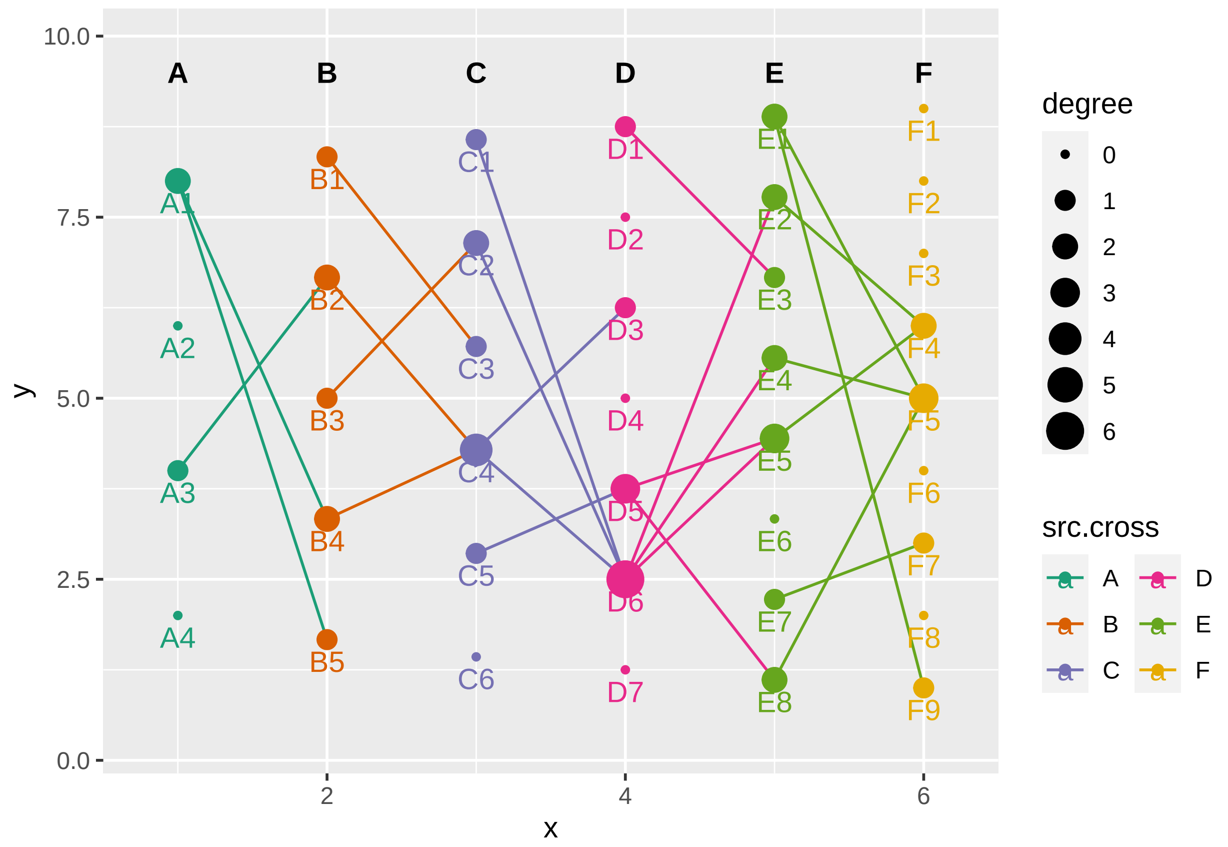


```
# set figure theme
# add:  other gg object to be added to final plot, such as theme().
theme_use <- theme(legend.position = "top", aspect.ratio = 1,
                   axis.title = element_blank(),
                   axis.text = element_blank(),
                   axis.ticks = element_blank(),
                   panel.grid = element_blank(),
                   panel.background = element_blank())

cl %>% cl_plot(add = theme_use)
```

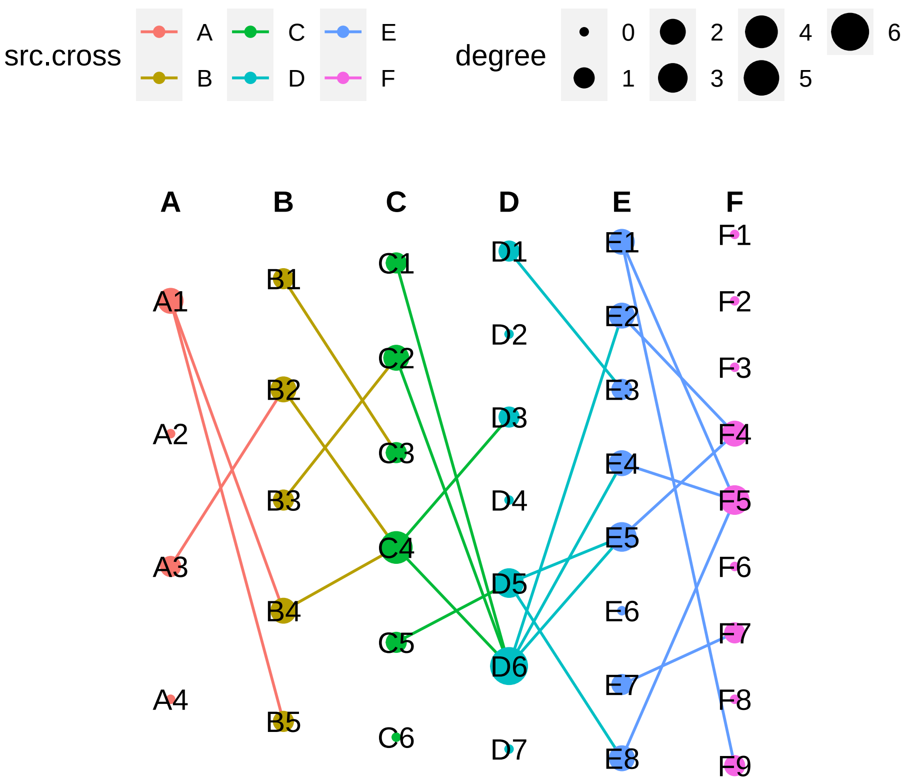


```
# combined all aesthetic settings.
cl %>% cl_plot(cross = list(mapping =  aes(color = type, shape= type),
                            scale   = list(color = scale_color_manual(values = RColorBrewer::brewer.pal
                                                                      guide = guide_legend(ncol = 2)),
                                           size  = scale_size_continuous(range = c(1,5),
                                                                      guide = guide_legend(ncol = 2)),
                                           shape  = scale_shape_manual(values = c(13:18),
                                                                      guide = guide_legend(ncol = 2))
                                          )
                           ),
               link = list(mapping =  aes(x = x + 0.1, xend = xend -0.1,
                                          color = src.cross,linetype =src.cross, size = src.degree),
                            scale   = list(color = scale_color_manual(values = RColorBrewer::brewer.pal
                                                                      guide = guide_legend(ncol = 2)),
                                           size  = scale_size_continuous(range = c(1,3),
                                                                      guide = guide_legend(ncol = 2)),
                                           linetype = scale_linetype_manual(values = c(1:6),
                                                                      guide = guide_legend(ncol = 2))
                                           ),
                            size = 1.5
                            ),
               header = list(mapping = aes(color= cross),
                               scale = list(color = scale_color_manual(values = RColorBrewer::brewer.pal
                                                                       guide = guide_legend(ncol = 2)))
                                size = 5.5
                             ),
```

```
                label = list(nudge_y = -0.3, size = 4
                            ),
                add = theme_use
                )
```

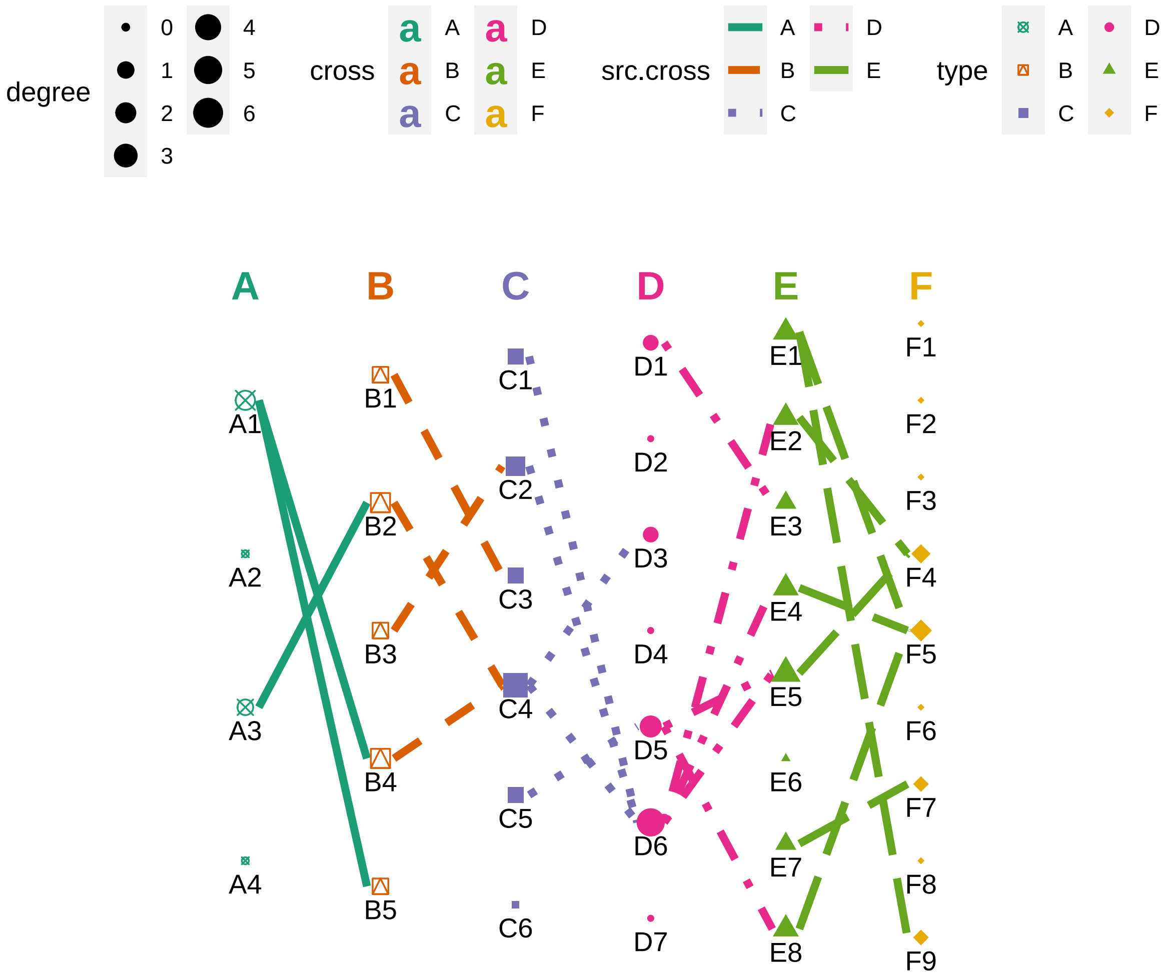

c. annotation figure

```
# cl_annotation  add annotation figure.
# top, bottom, left or  right   : ggplot object
# top.by, bottom.by, left.by, right.by : name of cross by which to align ggplot

ann.data <- data.frame(F=factor(paste0("F",c(1:10)),levels=paste0("F",c(10:1))),value=sample(size = 10,
ann.data %>% ggplot(mapping = aes(x=F,y=value))+geom_bar(stat="identity")+coord_flip() -> rgtAnn

cl %>% cl_plot(annotation=cl_annotation(right= rgtAnn,right.by ="F"))
```

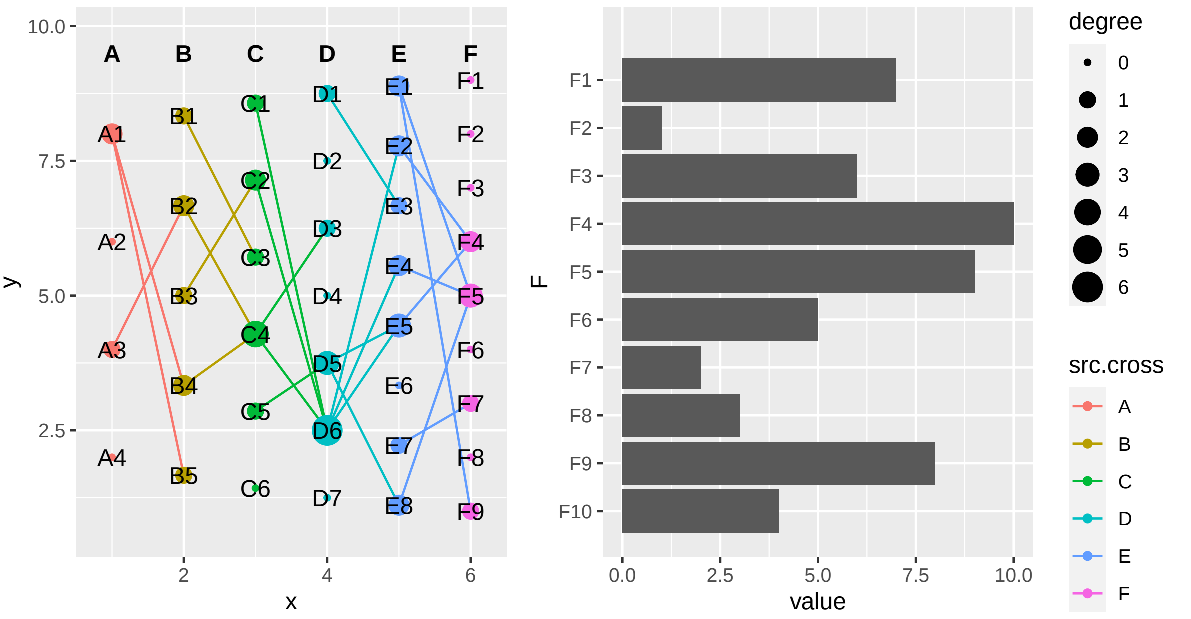

d. custom plots using ggplot2

Retrieving the metadata for nodes, edges and headers, with which users can plot the network in any way they like.

```
cl %>% get_cross()    # get node information
```

```
##      node node.type x        y cross key type degree
## A2    A4      node 1 2.000000     A  A4    A      0
## A3    A3      node 1 4.000000     A  A3    A      1
## A4    A2      node 1 6.000000     A  A2    A      0
## A5    A1      node 1 8.000000     A  A1    A      2
## B2    B5      node 2 1.666667     B  B5    B      1
## B3    B4      node 2 3.333333     B  B4    B      2
## B4    B3      node 2 5.000000     B  B3    B      1
## B5    B2      node 2 6.666667     B  B2    B      2
## B6    B1      node 2 8.333333     B  B1    B      1
## C2    C6      node 3 1.428571     C  C6    C      0
## C3    C5      node 3 2.857143     C  C5    C      1
## C4    C4      node 3 4.285714     C  C4    C      4
## C5    C3      node 3 5.714286     C  C3    C      1
## C6    C2      node 3 7.142857     C  C2    C      2
## C7    C1      node 3 8.571429     C  C1    C      1
## D2    D7      node 4 1.250000     D  D7    D      0
## D3    D6      node 4 2.500000     D  D6    D      6
## D4    D5      node 4 3.750000     D  D5    D      3
## D5    D4      node 4 5.000000     D  D4    D      0
## D6    D3      node 4 6.250000     D  D3    D      1
## D7    D2      node 4 7.500000     D  D2    D      0
## D8    D1      node 4 8.750000     D  D1    D      1
```

```
## E2    E8      node 5 1.111111     E  E8     E       2
## E3    E7      node 5 2.222222     E  E7     E       1
## E4    E6      node 5 3.333333     E  E6     E       0
## E5    E5      node 5 4.444444     E  E5     E       3
## E6    E4      node 5 5.555556     E  E4     E       2
## E7    E3      node 5 6.666667     E  E3     E       1
## E8    E2      node 5 7.777778     E  E2     E       2
## E9    E1      node 5 8.888889     E  E1     E       2
## F2    F9      node 6 1.000000     F  F9     F       1
## F3    F8      node 6 2.000000     F  F8     F       0
## F4    F7      node 6 3.000000     F  F7     F       1
## F5    F6      node 6 4.000000     F  F6     F       0
## F6    F5      node 6 5.000000     F  F5     F       3
## F7    F4      node 6 6.000000     F  F4     F       2
## F8    F3      node 6 7.000000     F  F3     F       0
## F9    F2      node 6 8.000000     F  F2     F       0
## F10   F1      node 6 9.000000     F  F1     F       0
```

```
cl %>% get_link()     # get edges information
```

```
##    src tar src.cross tar.cross source target src.degree tar.degree x        y xend
## 1   A1  B4         A         B     A1     B4          2          2 1 8.000000    2
## 2   A3  B2         A         B     A3     B2          1          2 1 4.000000    2
## 3   A1  B5         A         B     A1     B5          2          1 1 8.000000    2
## 4   B3  C2         B         C     B3     C2          1          2 2 5.000000    3
## 5   B2  C4         B         C     B2     C4          2          4 2 6.666667    3
## 6   B1  C3         B         C     B1     C3          1          1 2 8.333333    3
## 7   B4  C4         B         C     B4     C4          2          4 2 3.333333    3
## 8   C4  D3         C         D     C4     D3          4          1 3 4.285714    4
## 9   C5  D5         C         D     C5     D5          1          3 3 2.857143    4
## 10  C2  D6         C         D     C2     D6          2          6 3 7.142857    4
## 11  C4  D6         C         D     C4     D6          4          6 3 4.285714    4
## 12  C1  D6         C         D     C1     D6          1          6 3 8.571429    4
## 13  D6  E4         D         E     D6     E4          6          2 4 2.500000    5
## 14  D6  E5         D         E     D6     E5          6          3 4 2.500000    5
## 15  D1  E3         D         E     D1     E3          1          1 4 8.750000    5
## 16  D5  E8         D         E     D5     E8          3          2 4 3.750000    5
## 17  D6  E2         D         E     D6     E2          6          2 4 2.500000    5
## 18  D5  E5         D         E     D5     E5          3          3 4 3.750000    5
## 19  E2  F4         E         F     E2     F4          2          2 5 7.777778    6
## 20  E4  F5         E         F     E4     F5          2          3 5 5.555556    6
## 21  E5  F4         E         F     E5     F4          3          2 5 4.444444    6
## 22  E7  F7         E         F     E7     F7          1          1 5 2.222222    6
## 23  E8  F5         E         F     E8     F5          2          3 5 1.111111    6
## 24  E1  F9         E         F     E1     F9          2          1 5 8.888889    6
## 25  E1  F5         E         F     E1     F5          2          3 5 8.888889    6
##        yend
## 1  3.333333
## 2  6.666667
## 3  1.666667
## 4  7.142857
## 5  4.285714
## 6  5.714286
## 7  4.285714
```

```
## 8  6.250000
## 9  3.750000
## 10 2.500000
## 11 2.500000
## 12 2.500000
## 13 5.555556
## 14 4.444444
## 15 6.666667
## 16 1.111111
## 17 7.777778
## 18 4.444444
## 19 6.000000
## 20 5.000000
## 21 6.000000
## 22 3.000000
## 23 5.000000
## 24 1.000000
## 25 5.000000
```

```
cl %>% get_header()  # get header of crosslink object
```

```
##       node node.type x   y cross header
## 1 A_HEADER    header 1 9.5     A      A
## 2 B_HEADER    header 2 9.5     B      B
## 3 C_HEADER    header 3 9.5     C      C
## 4 D_HEADER    header 4 9.5     D      D
## 5 E_HEADER    header 5 9.5     E      E
## 6 F_HEADER    header 6 9.5     F      F
```

4. Examples

There are several examples and practical applications.

1). examples used in the paper

generate a CrossLink object

```
cl <- crosslink(demo$nodes, demo$edges, demo$cross.by, odd.rm = F,spaces = "flank")
cl %<>% set_header(header = c("A","B","C","D","E","F"))
```

a. layout by row

```
cl %>% layout_row() %>%
  set_header(hjust = 0, vjust = 0.5) %>%
   cl_plot(cross = list(mapping = aes(fill=type),
                        scale   = list(color = scale_color_manual(values = RColorBrewer::brewer.pal(8,
                                       fill = scale_fill_manual( values = RColorBrewer::brewer.pal(8,
                        size=8,shape=24, color = "black"
                       ),
          link   = list(mapping = aes(color = src.cross),
                        size=1.5, linetype=1),
```

```
          label  = list(color="white"),
          header = list(mapping = aes(color= cross),
                        scale = list(color = scale_color_manual(values = RColorBrewer::brewer.pal(8, "Da
                        size=5, show.legend = F
                       )
          )  %>%
  cl_void(th = theme(aspect.ratio = 1))
```

```
## Copy layout default into default, and Set active layout to default

## Copy layout temp into default, and Set active layout to default
```

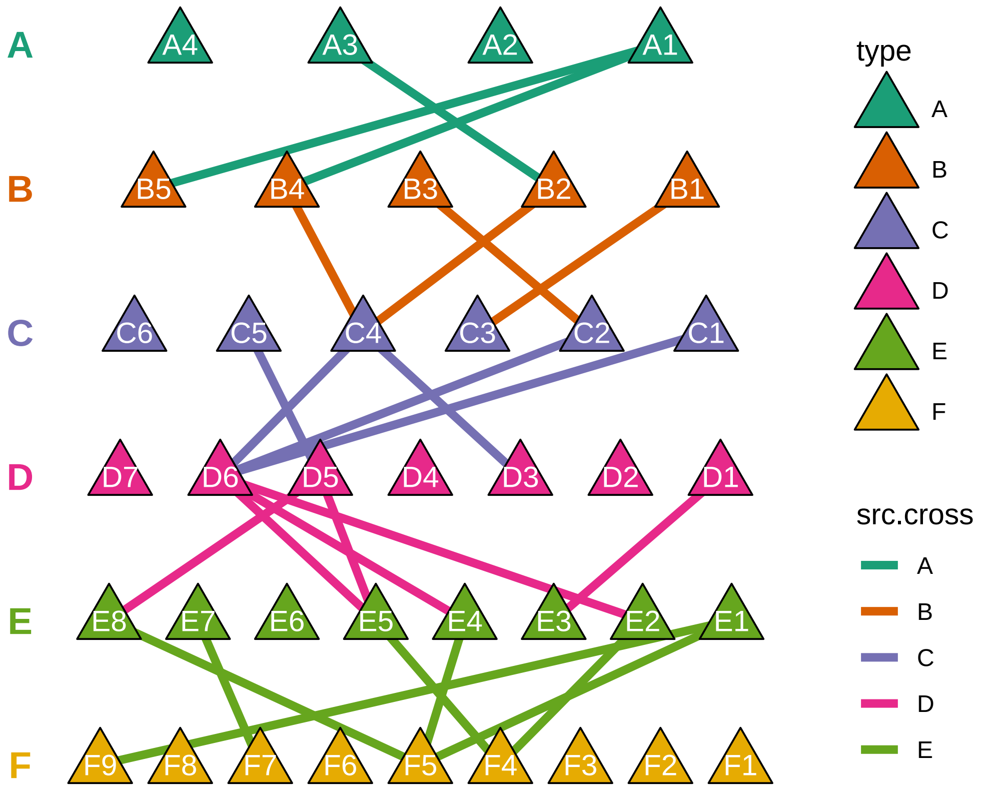

b. layout by rotate

```
cl %>%
   tf_rotate(angle = 10, by.each.cross = T) %>%
   cl_plot(cross = list(mapping = aes(fill = type),
                       scale = list(fill = scale_fill_manual(values = RColorBrewer::brewer.pal(8, "Dark2
                       size=10, shape=24, color = "black"
                       ),
          link   = list(mapping = aes(color = src.cross),
                        size=1.5, linetype=1),
          label = list(color="white"),
```

```
        header = list(mapping = aes(color= cross),
                      scale = list(color = scale_color_manual(values = RColorBrewer::brewer.pal(8, "Da
                      size=5, show.legend = F
                      )
        )  %>%
  cl_void(th = theme(aspect.ratio = 1))
```

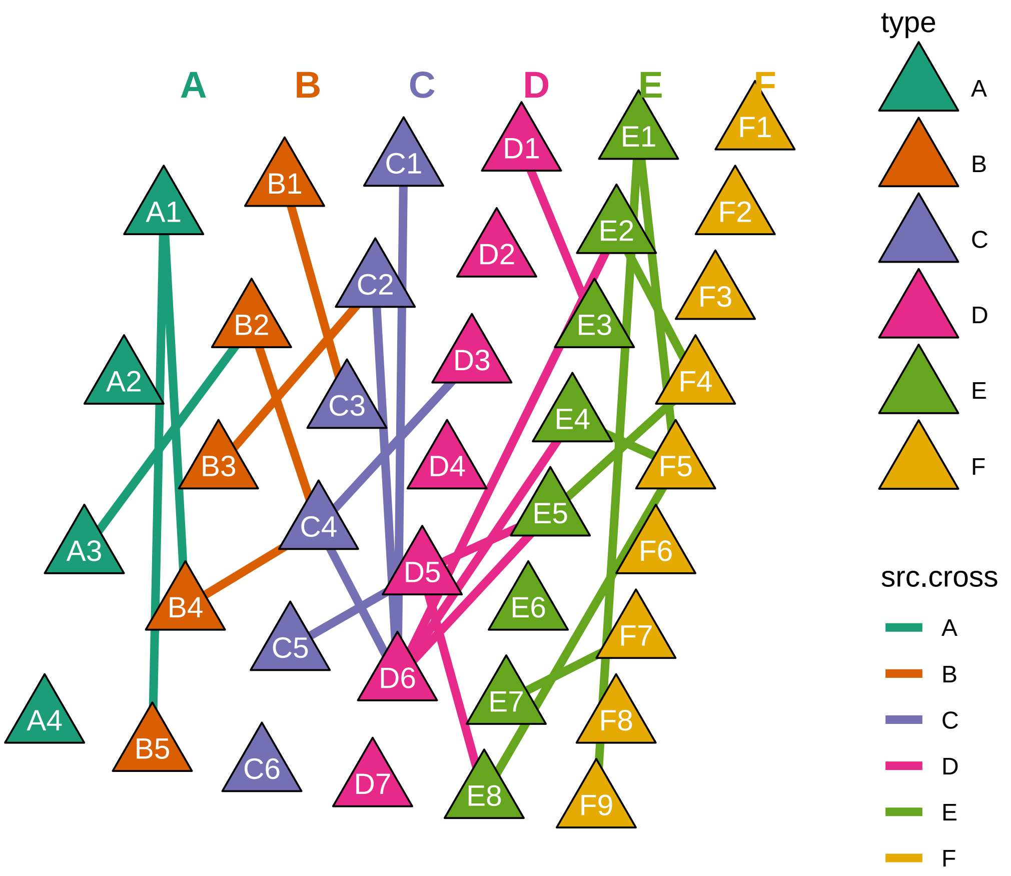

c. layout by polygon

```
cl %>% layout_polygon() %>%
   #tf_rotate(angle = 45, by.each.cross = F) %>%  # If the grouping is 4, rotate by this parameter and
   cl_plot(cross = list(mapping = aes(color = type),
                        scale = list(color = scale_color_manual(values = RColorBrewer::brewer.pal(8, "Da
                        size=10,shape=16
                       ),
           link   = list(geom = "curve",
                         mapping = aes(color = src.cross),
                         size=1.5, linetype=1
                         ),
           label = list(color="white"),
           header= list(mapping = aes(color= cross),
                        scale = list(color = scale_color_manual(values = RColorBrewer::brewer.pal(8, "Da
                        size=5
                        )
```

```
          )  %>%
  cl_void(th = theme(aspect.ratio = 1))
```

```
## Copy layout default into default, and Set active layout to default

## Copy layout temp into default, and Set active layout to default
```

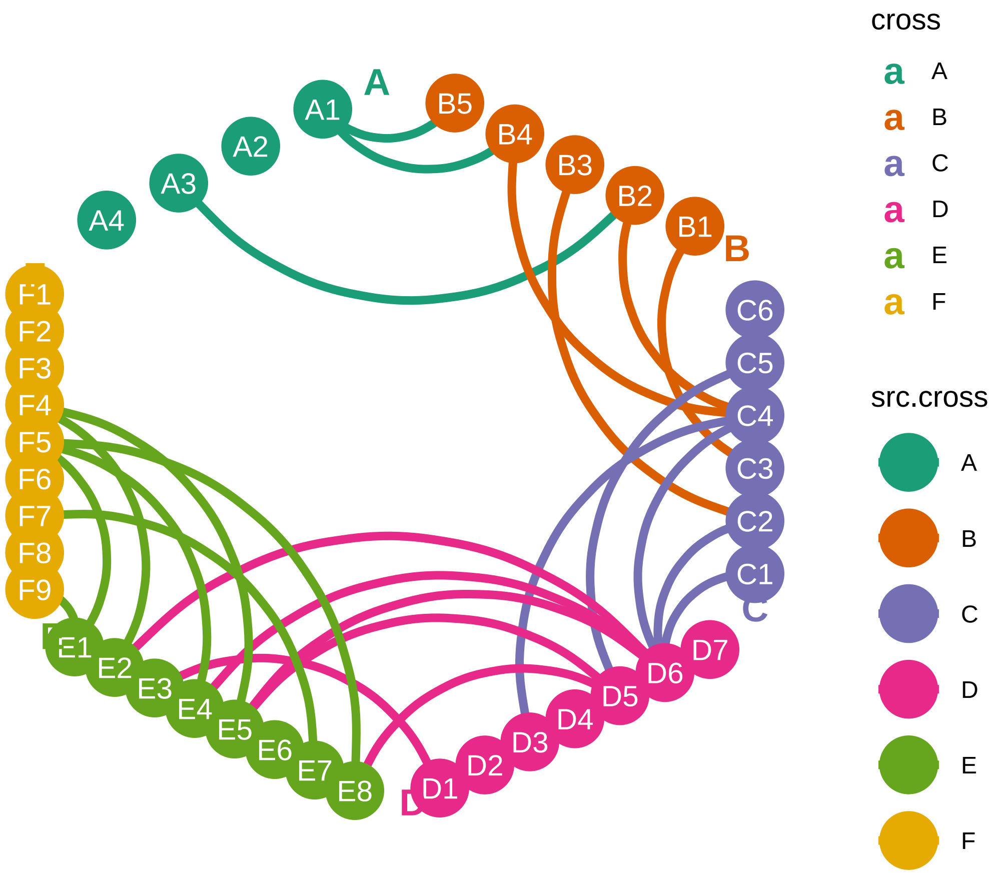

d. layout by hive

```
cl %>% layout_hive(angles=rep(60,6)) %>% #length of angles must be same with the numbers of groups
      cl_plot(cross = list(mapping = aes(color = type),
                           scale = list(color = scale_color_manual(values = RColorBrewer::brewer.pal(8, "Da
                           size=10,shape=18
                           ),
              link   = list(geom = "curve", curvature = -0.5,
                                 mapping = aes(color = src.cross),
                                 size=1.5, linetype=1),
              label = list(color="white"),
              header= list(mapping = aes(color= cross),
                                scale = list(color = scale_color_manual(values = RColorBrewer::brewer.pal(8
                                size=5
                                )
          )  %>%
  cl_void(th = theme(aspect.ratio = 1))
```

```
## Copy layout default into default, and Set active layout to default

## Copy layout temp into default, and Set active layout to default
```

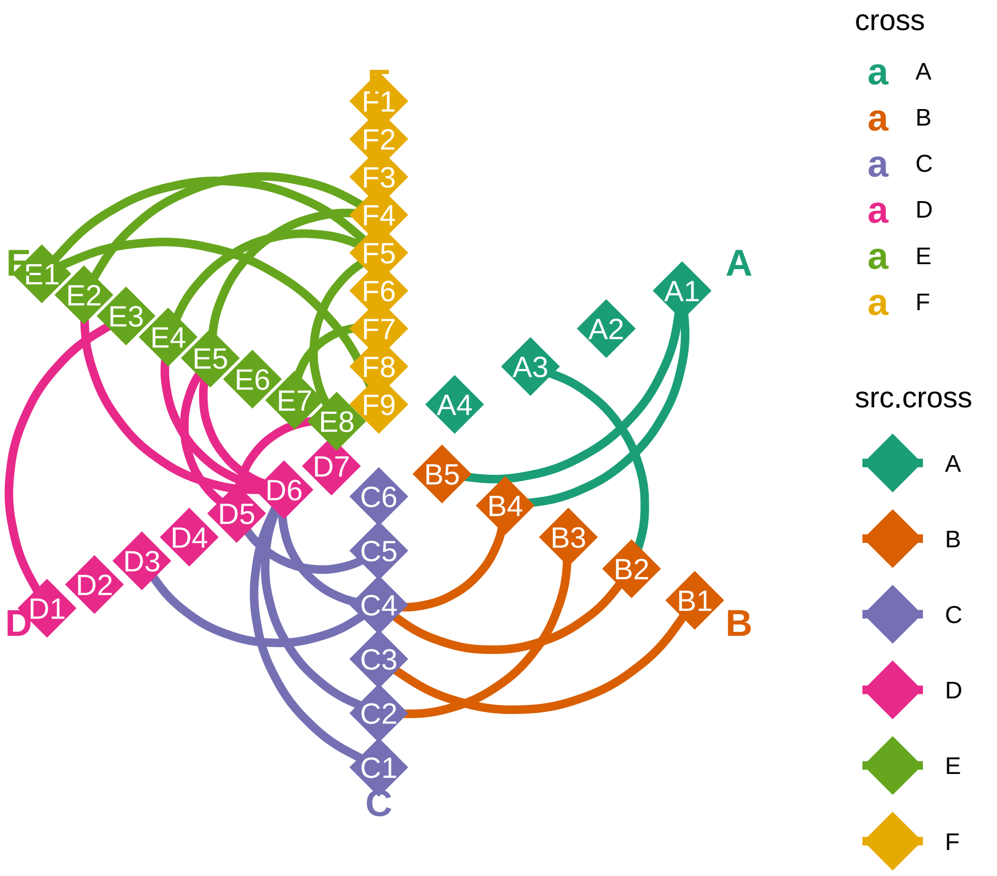

e. layout by arc

```
cl %>% layout_arc(angles = 45) %>% #length of angles must be same with the numbers of crosses
       cl_plot(cross = list(mapping = aes(color = type),
                            scale = list(color = scale_color_manual(values = RColorBrewer::brewer.pal(8
                            size=10, shape=18
                           ),
               link   = list(mapping = aes(color = src.cross),
                             size=1, linetype=2),
               label = list(color="white"),
               header= list(mapping = aes(color= cross),
                            scale = list(color = scale_color_manual(values = RColorBrewer::brewer.pal(8
                            size=5
                           )
               ) %>%
  cl_void(th = theme(aspect.ratio = 1))
```

```
## Copy layout default into temp, and Set active layout to temp

## Copy layout default into default, and Set active layout to default
```

```
## Copy layout temp into default, and Set active layout to default
```

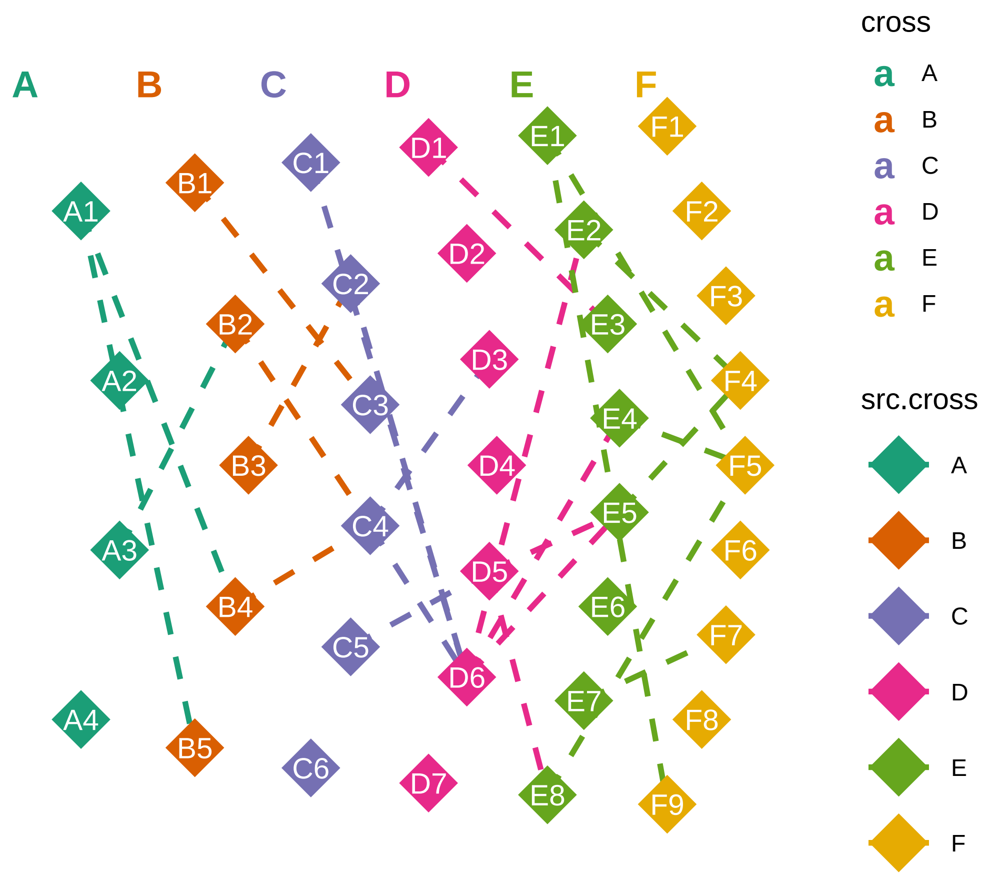

f. layout by combination of several methods

```
cl %>%
  layout_polygon(crosses = c("A","B","C","D"), layout_save = "combined") %>%
  layout_row(crosses = c("E", "F"), layout_save = "combined") %>%
  tf_rotate(crosses=c("A","B","C","D"), x = 0, y = 0, angle = -45) %>%
  tf_scale(crosses = c("E", "F"), x = 0.5, y = 0.5, scale.x = sqrt(2), scale.y = 3) %>%
  cl_align(crosses.1 = c("A","B","C","D"), crosses.2 = c("E", "F"),
           align.x = T, align.y = T,
           anchor.1 = c(0.5, 0),
           anchor.2 = c(0.5, 2)) %>%
   cl_plot(cross = list(mapping = aes(color = type),
                        scale = list(color = scale_color_manual(values = RColorBrewer::brewer.pal(8, "Da
                        size=10,shape=19
                        ),
          link   = list(color="grey75",size=1,linetype=3),
          label  = list(color="white"),
          header = list(mapping = aes(color= cross),
                        scale = list(color = scale_color_manual(values = RColorBrewer::brewer.pal(8, "Da
                        size=5
                        )
   ) %>%
  cl_void(th = theme(aspect.ratio = 1))
```

```
## Copy layout default into combined, and Set active layout to combined

## Copy layout temp into combined, and Set active layout to combined

## Copy layout combined into combined, and Set active layout to combined

## Copy layout temp into combined, and Set active layout to combined
```

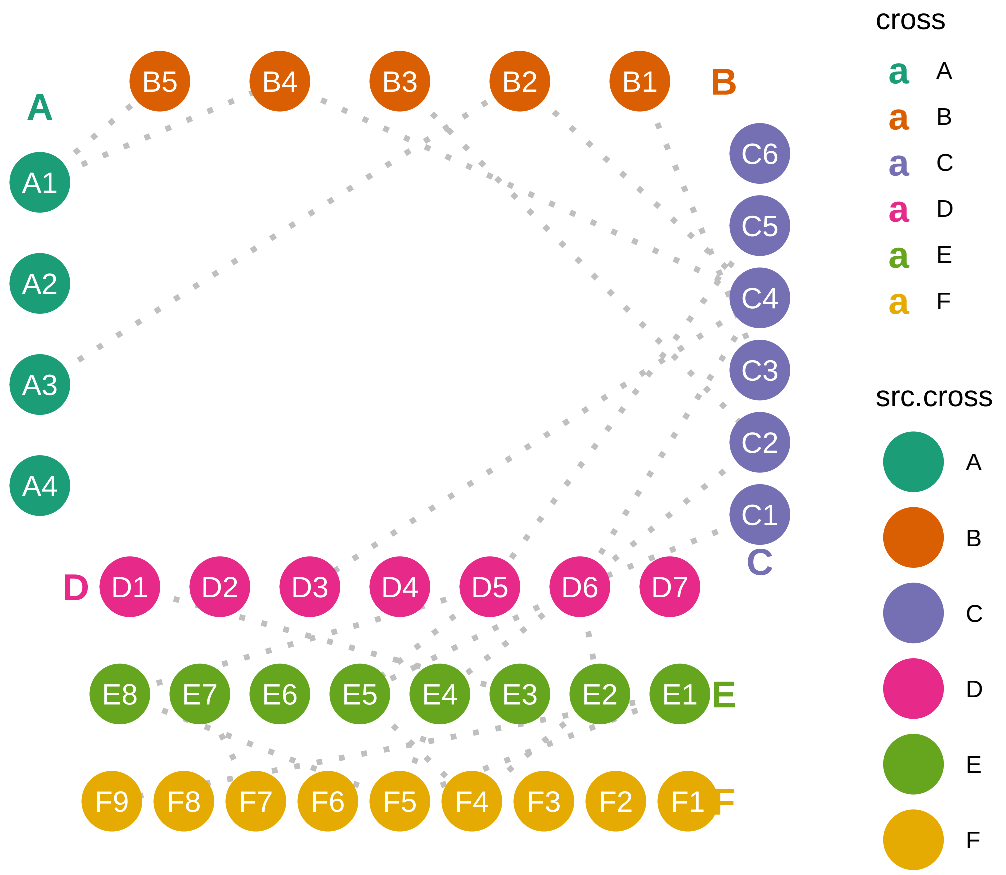

2). examples of complex figure

```
library(dplyr)
library(reshape)
theme_classic() +
  theme(axis.text = element_blank(),
        axis.line.x = element_blank(),
        axis.ticks.x = element_blank()) ->theme_use2


## crosslink project
cl <- crosslink(example$nodes,example$edges,cross.by="type")

cl %>% cl_plot()
```

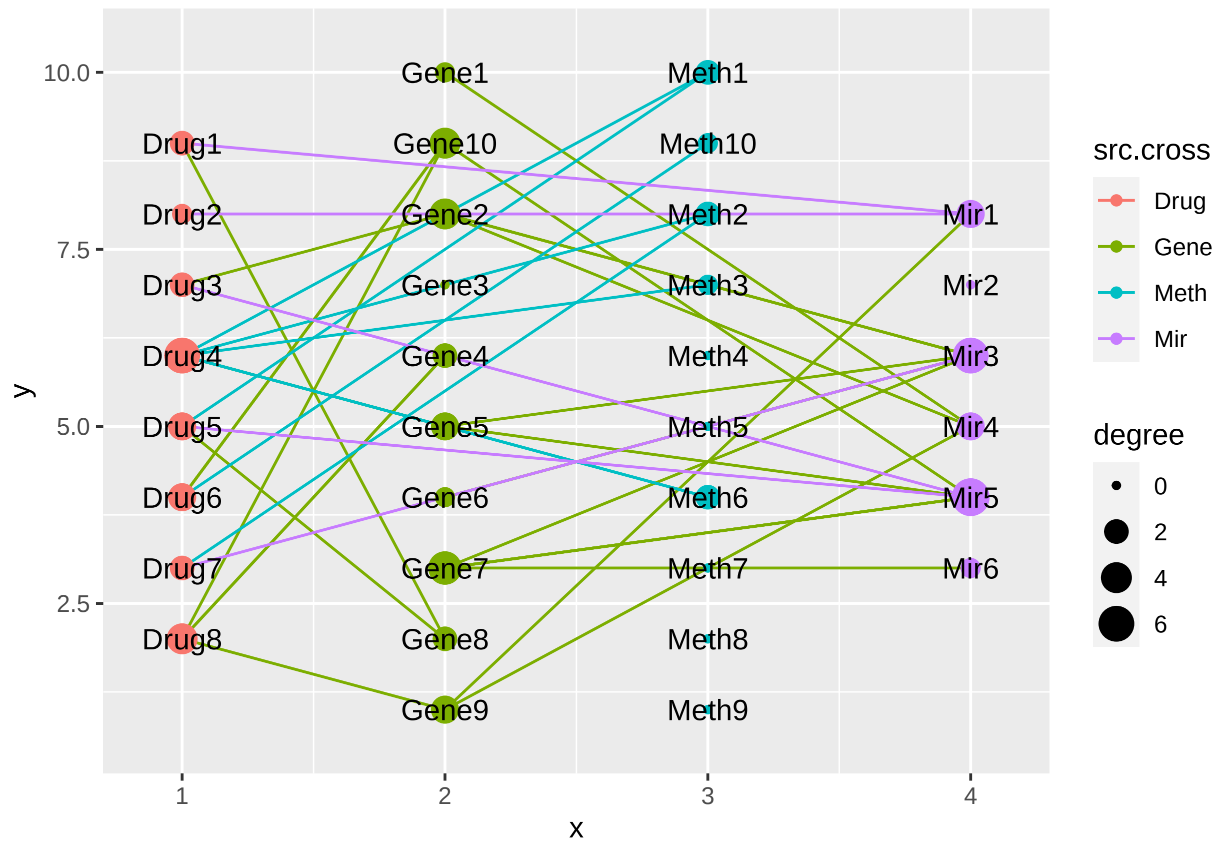


```
cl <- set_header(cl,header=unique(get_cross(cl)$cross))

cl %>% layout_polygon(crosses = c("Mir","Meth","Gene","Drug"),layout_based = "default") %>%
       tf_rotate(crosses= c("Mir","Meth","Gene","Drug"),angle = rep(45,4),layout="default") %>%
       tf_shift(x=0.2*(-1),y=1.5,crosses=c("Mir","Meth"),layout="default") -> cl
```

```
## Copy layout default into default, and Set active layout to default

## Copy layout temp into default, and Set active layout to default
```

```
# plot annotation
top <- nodes$id[nodes$type == "Mir"] # set the order as you like
bottom <- nodes$id[nodes$type == "Gene"] # set the order as you like
right <- nodes$id[nodes$type == "Meth"] # set the order as you like

# Top plot
topAnn <-  mirData %>%
  mutate(mir_f = factor(mir, top)) %>%
  ggplot(mapping = aes(x=mir,y=-lfc)) +
  geom_bar(fill = "#E7298A",
           stat = "identity",
           width = 0.5) +
  labs(x = NULL, y = "log2(Fold Change)") +
  theme_use2
topAnn
```

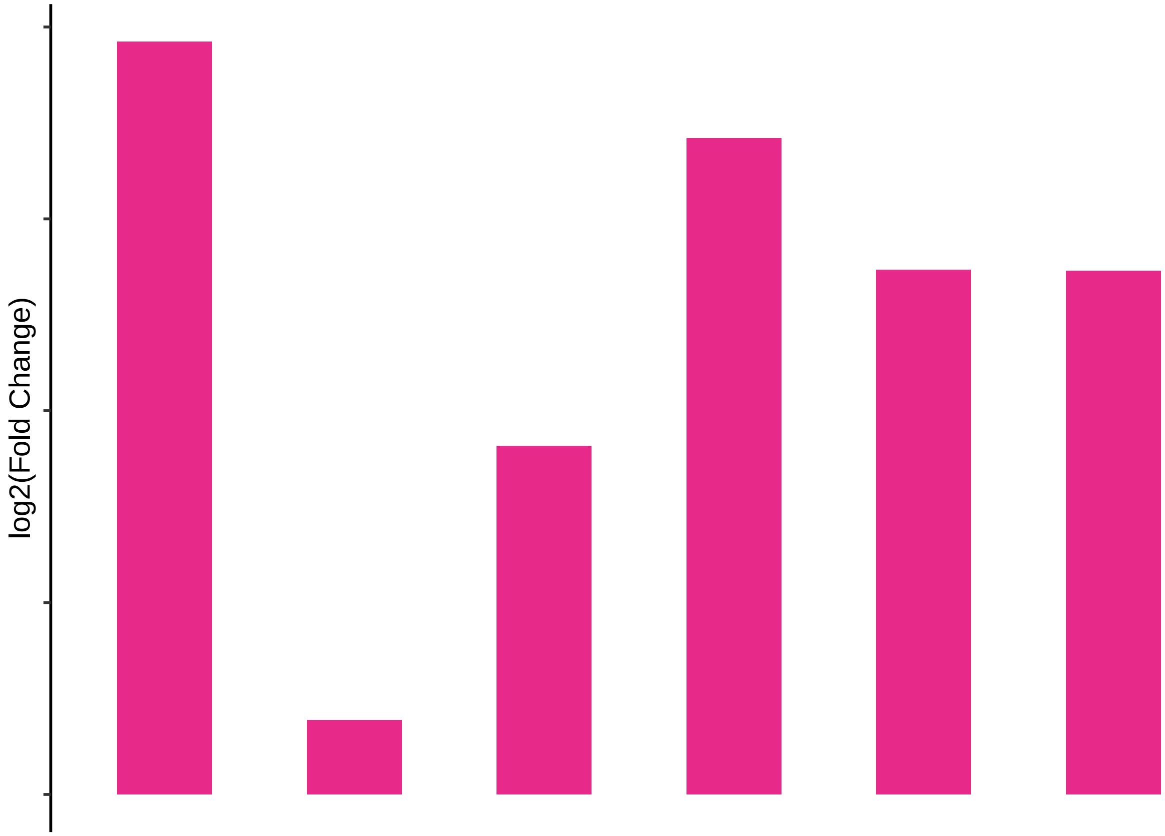


```r
# Bottom plot
botAnn <- geneData %>%
  mutate(meth_f = factor(gene, bottom)) %>%
  ggplot(mapping = aes(x=gene,y=-lfc)) +
  geom_bar(fill =  RColorBrewer::brewer.pal(8, "Dark2")[c(1:5,7:9)][2],
           stat = "identity",
           width = 0.5) +
  labs(x = NULL, y = "Difference") +
  theme_use2
botAnn
```

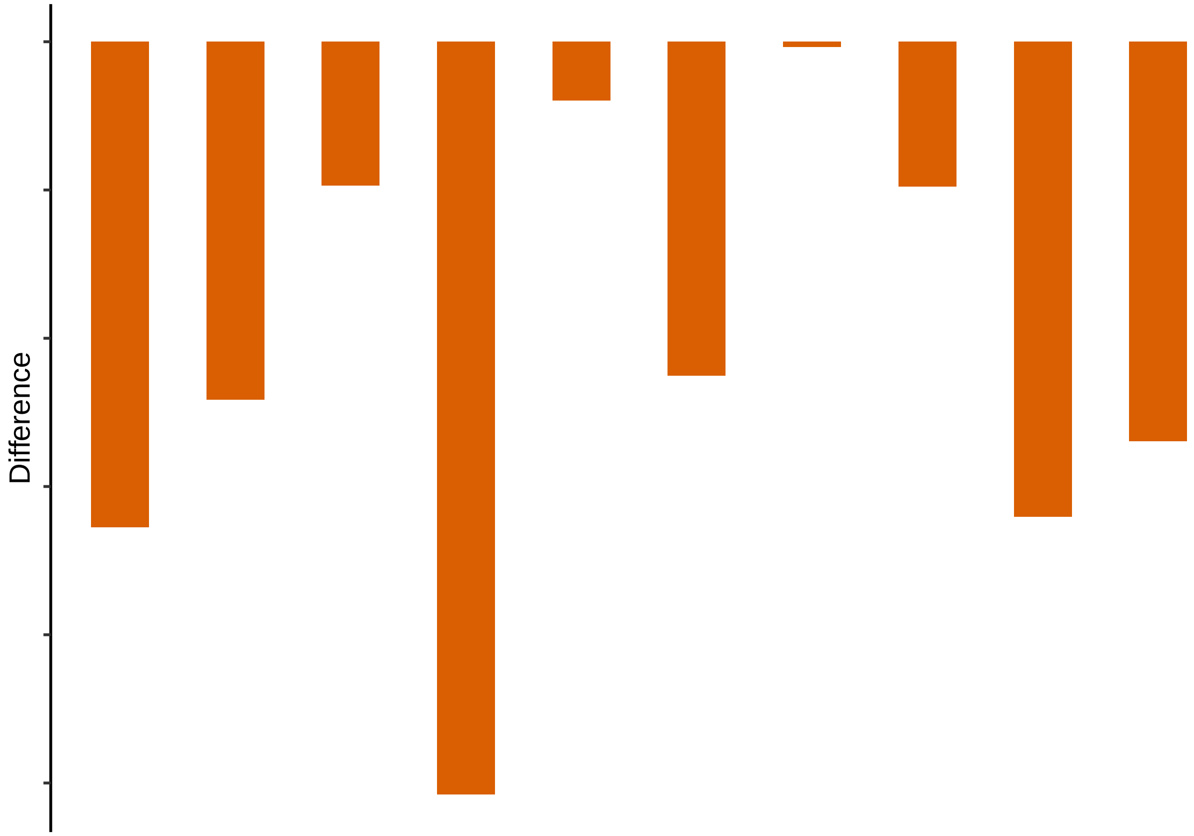


```r
# right plot
rgtAnn <- methData %>%
  mutate(mir_f = factor(meth, right)) %>%
  ggplot(mapping = aes(x=meth,y=-lfc)) +
  geom_bar(fill = RColorBrewer::brewer.pal(8, "Dark2")[c(1:5,7:9)][3],
           stat = "identity",
           width = 0.5) +
  labs(x = NULL, y = "log2(Fold Change)") +
  theme_use2 +
  coord_flip()

rgtAnn
```

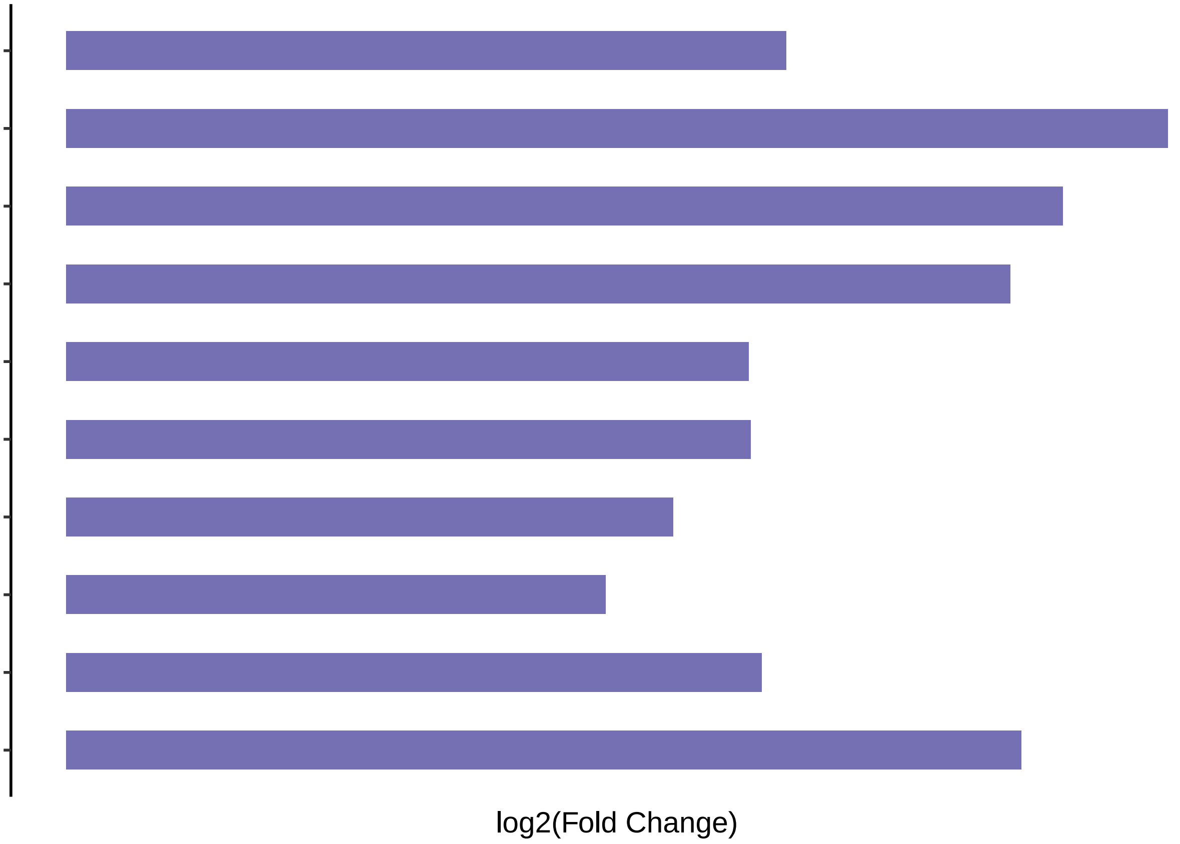


```r
# left plot
mat = matrix(sample(1:100, 64, replace = T), nrow = 8)
colnames(mat)=as.character(cl@cross$Drug)
ggplot(data = melt(mat), aes(X1, X2, fill = value))+
     geom_tile(color = "white")+
     scale_fill_gradient2(low = "blue", high = "darkgreen", mid = "white",midpoint = 0) +
     xlab("")+ylab("")+theme_classic()+
     theme(legend.position = "right",
          axis.line = element_blank(),
          axis.ticks = element_blank(),
           axis.text = element_blank()) -> lftAnno
lftAnno
```

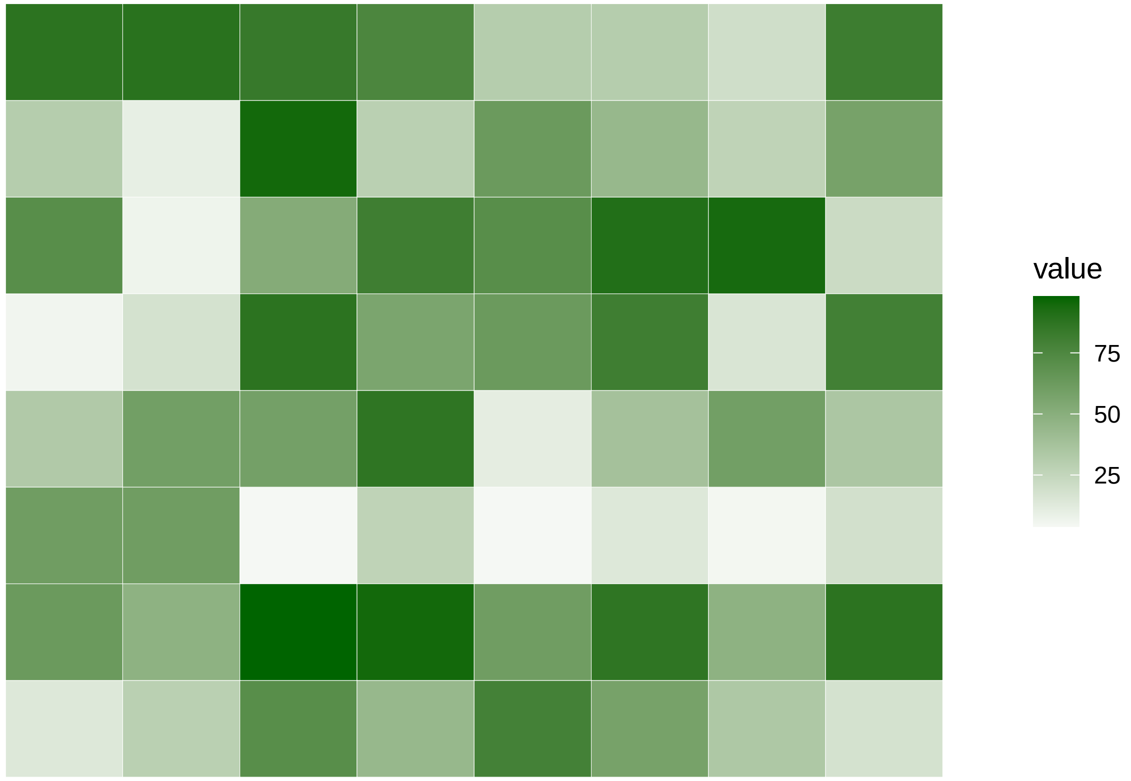

Combine network plot and four annotation plots

```
cl_plot(cl,
       annotation=cl_annotation(top = topAnn,top.by = "Mir",top.height = 0.5,
                                bottom = botAnn,bottom.by = "Gene",bottom.height = 0.5,
                                right = rgtAnn,right.by ="Meth",right.width = 0.5,
                                left = lftAnno,left.by = "Drug" ,left.width = 0.5),
       cross = list(mapping = aes(color = type,size=degree,shape=type),
       scale = list(color = scale_color_manual(values = RColorBrewer::brewer.pal(8, "Dark2")[c(1:5,7:9)[illegible]
                    shape = scale_shape_manual(values = 15:23),
                    size  = scale_size_continuous(range=c(1,5)))),
       link  = list(mapping = aes(color = type,linetype=type,size=cor),
                    scale = list(color = scale_color_manual(values = RColorBrewer::brewer.pal(8, "Set1")[illegible]
                                 linetype=scale_linetype_manual(values = c(1:4)),
                                 size=scale_size(range = c(1,2)))),
       header=NA,
       label = list(color="black"
                    ,angle=c(rep(0,8),rep(90,10),rep(0,10),rep(90,6))
                    ,nudge_x=c(rep(-2,8),rep(0,10),rep(2,10),rep(0,6))
                    ,nudge_y=c(rep(0,8),rep(-2,10),rep(0,10),rep(2,6))),
       add   =  theme(panel.background = element_blank(),
                      axis.title = element_blank(),
                      panel.grid = element_blank(),
                      axis.ticks = element_blank(),
                      axis.text = element_blank()))
```

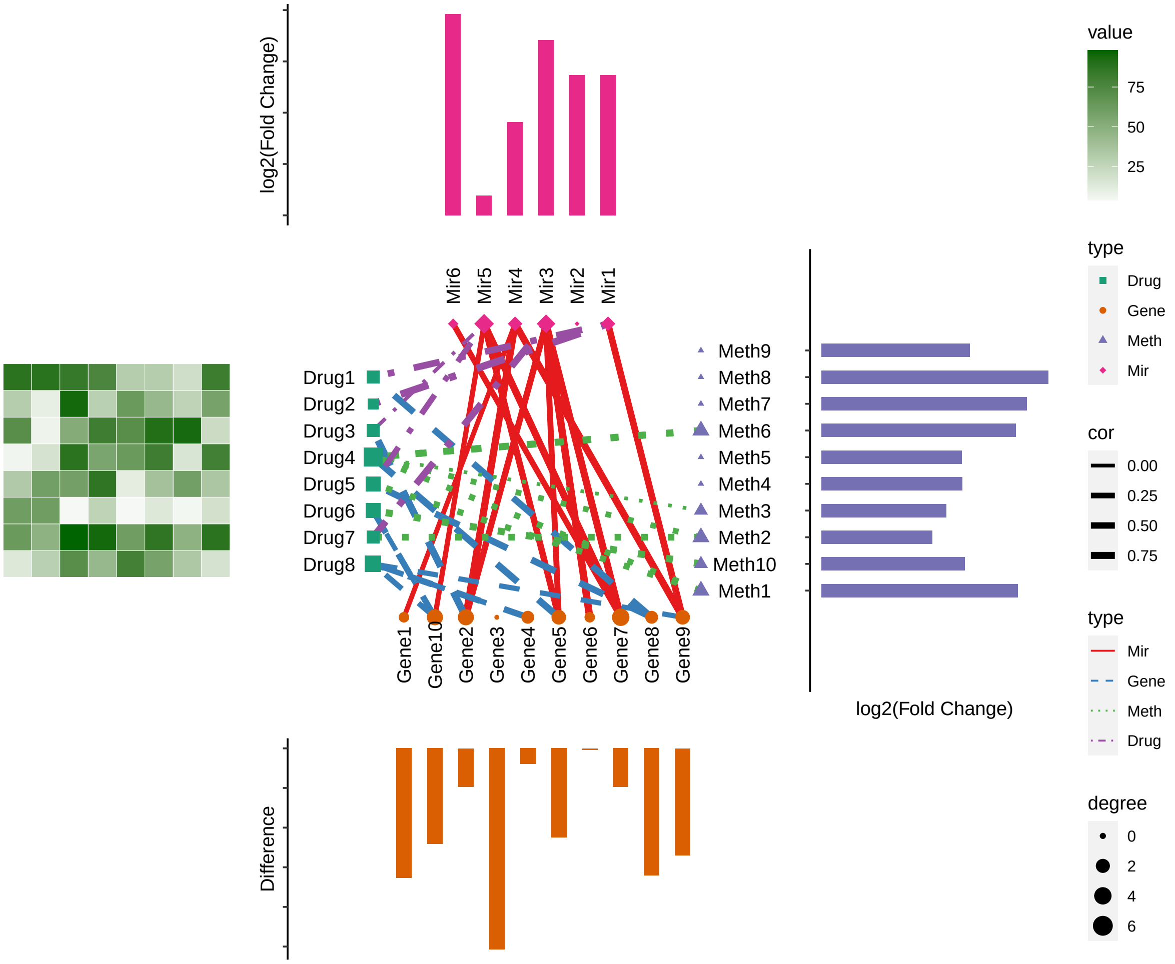

log2(Fold Change)
Mir6
Mir5
Mir4
Mir3
Mir2
Mir1
Drug1
Drug2
Drug3
Drug4
Drug5
Drug6
Drug7
Drug8
Meth9
Meth8
Meth7
Meth6
Meth5
Meth4
Meth3
Meth2
Meth10
Meth1
Gene1
Gene10
Gene2
Gene3
Gene4
Gene5
Gene6
Gene7
Gene8
Gene9
log2(Fold Change)
Difference
value
75
50
25
type
Drug
Gene
Meth
Mir
cor
0.00
0.25
0.50
0.75
type
Mir
Gene
Meth
Drug
degree
0
2
4
6
